# QTL for spike-layer uniformity and their influence on yield-related traits in wheat

**DOI:** 10.1186/s12863-019-0730-3

**Published:** 2019-02-28

**Authors:** Chunhua Zhao, Na Zhang, Yongzhen Wu, Han Sun, Cheng Liu, Xiaoli Fan, Xuemei Yan, Hongxing Xu, Jun Ji, Fa Cui

**Affiliations:** 1 College of Agriculture, Ludong University, Yantai, 264025 Shandong China; 2 Center for Agricultural Resources Research, Institute of Genetics and Developmental Biology, Chinese Academy of Sciences, Shijiazhuang, 050022 China; 3 Crop Research Institute, Shandong Academy of Agricultural Sciences, Jinan, 250100 China; 4 Chengdu Institute of Biology, Chinese Academy of Sciences, Chengdu, 610041 China; 5 Anqiu Municipal Burean of Agriculture, Anqiu, 262100 China; 6 Institute of Plant Stress Biology, State Key Laboratory of Cotton Biology, Department of Biology, Henan University, Kaifeng, 475001 China

**Keywords:** Quantitative trait locus, Recombinant inbred line, Spike-layer uniformity, Wheat, Yield

## Abstract

**Background:**

Common wheat (*Triticum aestivum* L.) is one of the most important food crops worldwide. Wheat spike-layer uniformity related traits (SLURTs) were complex traits that directly affect yield potential and appearance. In this study, quantitative trait locus (QTL) for five SLURTs among inter-tillers were first documented using a recombinant inbred line (RIL) mapping population derived from a cross between Kenong9204 and Jing411 (represented by KJ-RILs). Genetic relationships between SLURTs and yield were characterized in detail.

**Results:**

The trait phenotypic performances for the 188 KJ-RILs and their parents were evaluated in eight different environments. The genetic data included in a high-density genetic map derived from the Affymetrix 660 K SNP Array and the corresponding genotypes in each lines. Of 99 putative additive QTL 11 were stable across environments and 57 showed significant additive-by-environment interaction effects. These QTL individually explained 1.05–39.62% of the phenotypic variance, with log of odds (LOD) values ranging from 2.00 to 34.01. Genetic relationships between SLURTs and yield indicated that plants with slight uneven spike spatial distribution should be an ideotype for super high-yield in wheat.

**Conclusions:**

The present study will provide assistance in understanding the genetic relationships between SLURTs and yield potential. The 11 stable QTL for SLURTs identified herein may facilitate breeding new wheat varieties with scientifically reasonable spike-layer distribution by marker assisted selection.

**Electronic supplementary material:**

The online version of this article (10.1186/s12863-019-0730-3) contains supplementary material, which is available to authorized users.

## Background

Wheat (*Triticum aestivum* L.) is one of the most important food crops worldwide. Like rice, wheat is a crop with tillers. The main stem and tillers develop at different stages and display difference of the photosynthetic characteristics, resulting in differences in tiller heights among them [1]. Characterstics of the spike spatial distribution, i.e., spike-layer uniformity (SLU), were generally determined by variation of spike heights among inter-plants and among inter-tillers, which are two indexes of the population uniformity of wheat and rice [1, 2]. Of these, the SLU determined by differences among inter-plant heights was prone to be affected by environmental factors such as agronomic management. The SLU influenced by inter-tiller height difference is mostly determined by genotypes [2–8]. Varieties with consistent spike heights (generally regarding as good SLU) can not only please farmers but also facilitate mechanical harvest [5].

Genetic relationships between SLU and yield in wheat are complex. To date, limited studies focused on this scientific issues [5, 6, 9]. Yao [5] and Hu [6] proved that SLU had significantly negative correlation with yield potential; a plant with a slight difference in spike heights among tillers tends to own high yield potential. In contrast, Wang et al. [9] found that SLU was irrelevant with yield potential using two authorized wheat varieties as plant materials. No more relevant reports regarding SLU have been found during the last 15 years. In rice, fewer tillers and large panicles are admirable plant types suggested by International Rice Research Institute (IRRI) [10–12]. Less panicles is more beneficial to the uniformity of panicle layer, which was regarded as the ideotype plant with high yield potential in rice [1].

The large genome size (17 gigabases), hexaploid nature (AABBDD), and numerous repetitive DNA sequences of the genome have resulted in the relatively slow pace of wheat genomics research. However, great progresses have been made in wheat genomics research especially in the recent 5 years [13–19]. Benefiting from the wheat genomics research, high-density genetic map and numerous QTL for wheat plant type such as plant height [20–22] have been documented. However, no QTL for characteristics of wheat spike spatial distribution related traits has been documented by now.

The characteristics spike spatial distribution not only affect the appearance but also influence yield potential. In this study, QTL for six SLU related traits (SLURTs) were first documented based on a high-density map constructed from the Affymetrix 660 K SNP Array [23]. Genetic relationships between SLURTs and yield were also characterized. The objectives of this study were to 1) reveal the genetic characteristics of SLURTs in wheat; 2) specify the genetic relationships between SLURTs and yield related traits; 3) identify QTL for SLURTs in different environments; and 4) provide useful information for marker assisted selection (MAS) in breeding new wheat varieties with scientifically reasonable spike-layer distribution.

## Methods

### Experimental materials and evaluation

A recombinant inbred line (RIL) population comprising 188 lines derived from a cross between Kenong9204 (KN9204) and Jing411 (J411) (represented by KJ-RILs) was used in this study [22–25]. All the materials used in this study were provided by Center for Agricultural Resources Research, Institute of Genetics and Developmental Biology, Chinese Academy of Science. The absolute value of SLU of J411 was higher than that of KN9204. The 188 KJ-RILs and their parents were evaluated in eight environments as follows: E1 represents 2013–2014 in Shijiazhuang (37°53′N, 114°41′E, altitude 54 m) with normal nitrogen treatment (HN), E2 represents 2014–2015 in Shijiazhuang with HN, E3 represents 2013–2014 in Shijiazhuang with low nitrogen treatment (LN), E4 represents 2014–2015 in Shijiazhuang with LN, E5 represents 2016–2017 in Yantai (37°53′N, 121°37′E, altitude 4 m) with HN, E6 represents 2017–2018 in Yantai with HN, E7 represents 2016–2017 in Yantai with LN, and E8 represents 2017–2018 in Yantai with LN, respectively. Data were pooled across trials by calculating the average value of the corresponding traits in the above 8 environments, which was defined as the *P* trial.

The soil nitrate-nitrogen contents within the 0–20 cm layer in each environments are shown in (Additional file 1: Table S1). In each HN plot, 300 kg ha^− 1^ of diamine phosphate and 150 kg ha^− 1^ of urea were applied before sowing, and 150 kg ha^− 1^ of urea were applied at the elongation stage every year. In the LN plots, no N fertilizer (N-deficient) was applied throughout the growing period. A randomized complete block design (RCBD) with two replications was used in each of the eight environments, and 40 seeds were sown in each 2 m long rows spaced 25 cm. All recommended agronomic practices were followed in each of the trials except for the fertilization treatment as described above.

**Fig. 1:**
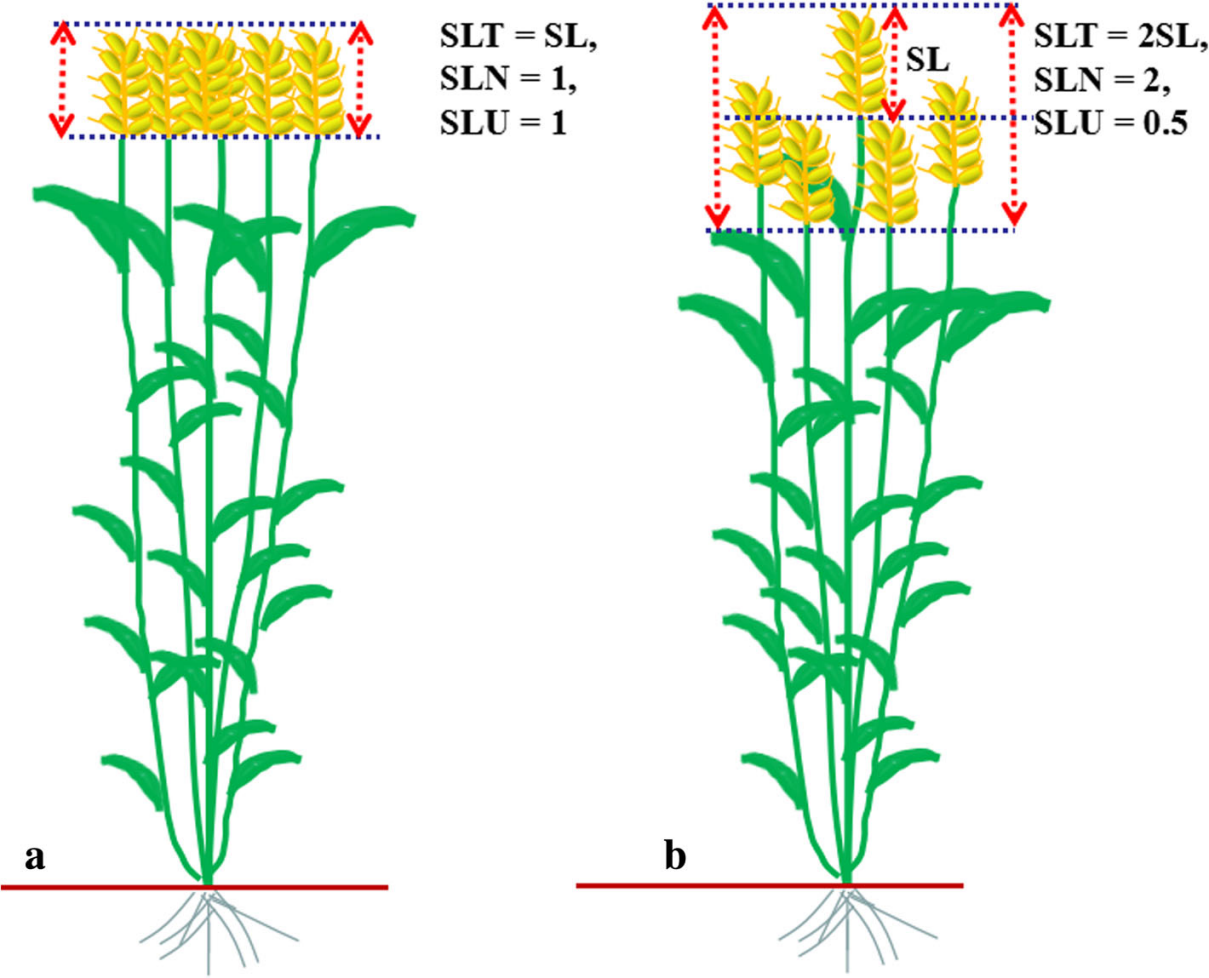
Biological meaning of spike-layer thickness (SLT), spike-layer number (SLN) and spike-layer uniformity (SLU). SLT indicated the spike layer thickness among inter-tillers; SLN indicated the number of spike layer per SLT; SLU indicated the consistency of the spike distribution in the vertical space. **a** All tillers per plant have identical tiller height, the SLT was identical to one spike length (SL) (SLT = SL); we can only see one spike layer from the vertical perspective (SLN = 1); all spikes have consistent vertical distribution (SLU = 1). **b** Most tillers per plant have different tiller height, the SLT was identical to two spike length (SL) (SLT = 2SL); we can see two spike layer from the vertical perspective (SLN = 2); spikes per plant have inconsistent vertical distribution (SLU = 0.5). The variation range of SLU should be from 0 to 1. Clearly, a larger SLU indicates a relative consistent spike vertical distribution

Three direct SLURTs were evaluated in this study, which were plant height (PH), the lowest tillers height (LTH) and spike length (SL). The highest tiller of each line was used to evaluate PH, measured from ground level to the tip of the spike, excluding awns. The lowest tiller with normal spike (with normal seed setting percentage and seed size) of each line were selected for evaluating LTH, measured from ground level to the tip of the spike, excluding awns. SL was measured from the base of the spike to the tip, excluding awns. Spike-layer thickness (SLT) was calculated as *SLT* = *PH*–*LTH + SL*; Spike-layer number (SLN) was calculated as *SLN*=SLT/SL; Spike-layer uniformity (SLU) was calculated as *SLU*=SL/SLT. The biological meaning of these novel indirect traits can be seen in Fig. 1. For each line, the main tillers of five plants were randomly selected from the middle of the row to measure the phenotype. The detailed information about the four yield-related traits (YRTs) including thousand-kernel weight (TKW), kernel number per spike (KNPS), spike number per plant (SNPP) and yield per plant (YPP) have been documented in our previous reports [22–25].

### Data analysis and QTL mapping

Basic statistical analysis of the phenotypic data in the KJ-RIL population among the eight environments was performed using the SPSS13.0 software (SPSS, Chicago, IL, USA; http:// en.wikipedia.org/wiki/SPSS). To evaluate the effects of genotype on SLURTs, Pearson correlations of the six SLURTs among environments in the KJ-RIL population were calculated. To evaluate the effects of SLURTs on YRTs, Pearson correlations between SLURTs and YRTs were calculated. The trait means for the eight environments were used to calculate phenotypic correlation coefficients between SLURTs and YRTs.

The high-density linkage map of the KJ-RIL included 5023 loci individually representing each bin on the 21 wheat chromosomes, which have been described in our previous reports [23]. Molecular markers of single-nucleotide polymorphism (SNP), simple sequence repeats (SSR), inter-simple sequence repeat (ISSR), sequence-tagged sites (STS) and sequence-related amplified polymorphism (SRAP) were included in the current genetic map. The corresponding marker information can be seen in our previous reports [23–25]. Markers were removed if they showed minor allele frequency (defined as frequency < 0.3) or contained > 10% missing data. A total of 10.4% of the 5023 mapped loci showed segregation distortion. The phenotypic values of the 188 KJ-RILs in E1, E2, E3, E4, E5, E6, E7, E8 and P were used for individual environment QTL mapping analysis. Only the QTL position for SL that co-located with SLURTs were shown in this report, whereas the detailed information will be documented in another paper. The inclusive composite interval mapping performed with IciMapping 4.1 (http://www.isbreeding.net/) was used to detect putative additive QTL. The walking speed chosen for all QTL was 1.0 cM, and the *P*-value inclusion threshold was 0.001. The threshold for the detection of QTL was calculated using 1000 permutations, with a type 1 error of 0.05. To detect QTL with significant additive-by-environment (A by E) interaction effects, combined (C) QTL analysis across environments was conducted, using 3.0 as the threshold LOD score. All of the QTL were designated according to Cui et al. [23]. A QTL with an average LOD value > 3.0 and an average phenotypic variance contribution > 10% was defined as a major QTL, and one showing significance in at least five of the nine data sets was considered as a stable QTL. We defined a QTL with significant A by E interaction effect when its LOD value for A by E is ≥2.5. A suggestive QTL with an average LOD value > 2.0 in a data set was shown and characterized if this QTL were significant in at least one of the rest data sets.

## Results

### Phenotypic performance of the six spike-layer uniformity related traits in the two parents and 188 KJ-RILs

Among the eight environments, J411 had significant higher values in LTH, PH, SL and SLU than KN9204 (*P* < 0.01). Conversely, KN9204 had higher values in SLN and SLT than J411 (*P* < 0.05), confirming that spikes of J411 had more consistent vertical spatial distribution than those of KN9204 (Additional file 1: Table S2). In the KJ-RIL population, the seven SLURTs exhibited approximately continuous variation in each environment. PH and SL were normally distributed in the KJ-RIL population in all the eight environments as well as in the P data set. LTH, SLN and SLT were approximately normally distributed in the KJ-RIL population in most cases, whereas the SLU showed deviation distribution in most cases (Additional file 1: Table S3).

The estimated broad-sense heritability of the six SLURTs ranged from 21.0% (for SLU) to 85.5% (for PH). The three directly measured traits (LTH, PH, SL) had higher level of broad-sense heritability than those of the compound traits, i.e., indirect measured traits such as SLT, SLN and SLU (Additional file 1: Table S3). Correlation coefficients of LTH, PH and SL among environments were shown in Additional file 1: Tables S4, S5 and S6, which were all significant across environments. SLT, SLN and SLU were significant across environments in most but not all of the cases (Additional file 1: Tables S7, S8 and S9).

The phenotypic correlations among the six traits are listed in Table 1. Significant positive correlations were observed between LTH and PH, LTH and SL, LTH and SLN, LTH and SLT, PH and SL, PH and SLU, PH and SLN, PH and SLT, and between SLN and SLT. SLU had significant and negative correlation with LTH, PH, SLN and SLT. SL and SLN had significant and negative correlation with each other at the *P* < 0.05 level.

**Table 1:** Phenotypic correlation coefficients between six spike layer uniformity related traits based on the averaged trait value among the eight environments

	LTH	PH	SL	SLU	SLN	SLT
LTH	1					
PH	0.95^**^	1				
SL	0.25^**^	0.34^**^	1			
SLU	−0.22^**^	− 0.46^**^	0.14	1		
SLN	0.21^**^	0.46^**^	− 0.16^*^	−0.99^**^	1	
SLT	0.35^**^	0.62^**^	0.55^**^	− 0.74^**^	0.73^**^	1

*LTH* the lowest tillers height, *PH* plant height, *SL* spike length, *SLU* spike-layer uniformity, *SLN* spike-layer number, *SLT* spike-layer thickness

^**^represents that correlation is significant at when *P* < 0.01 level; ^*^ represents that correlation is significant at when *P* < 0.05 level

Pearson correlations between SLURTs and YRTs were shown in Table 2. PH, LTH, SLN and SLT had significant and positive correlation with TKW and YPP; whereas the SLU had significant and negative correlation with TKW and YPP. All the six SLURTs had little effects on KNPS and SNPP.

**Table 2:** Phenotypic correlation coefficients between spike layer uniformity related traits and yield related traits in the 188 KJ-RILs among the nine environments

	PH	LTH	SLN	SLT	SLU
TKW	0.52^**^	0.53^**^	0.31^**^	0.17^*^	−0.31^**^
KNPS	− 0.06	− 0.09	−0.12	0.10	0.11
SNPP	−0.13	−0.18^*^	0.04	0.07	−0.04
YPP	0.28^**^	0.21^**^	0.21^**^	0.30^**^	− 0.23^**^

*LTH* the lowest tillers height, *PH* plant height, *SL* spike length, *SLU* spike-layer uniformity, *SLN* spike-layer number, *SLT* spike-layer thickness, *TKW* thousand-kernel weight, *KNPS* kernel number per spike, *SNPP* spike number per plant, *YPP* yield per plant

^**^represents that correlation is significant at when *P* < 0.01 level; ^*^ represents that correlation is significant at when *P* < 0.05 level

### QTL for the five spike-layer uniformity related traits

**Fig. 2:**
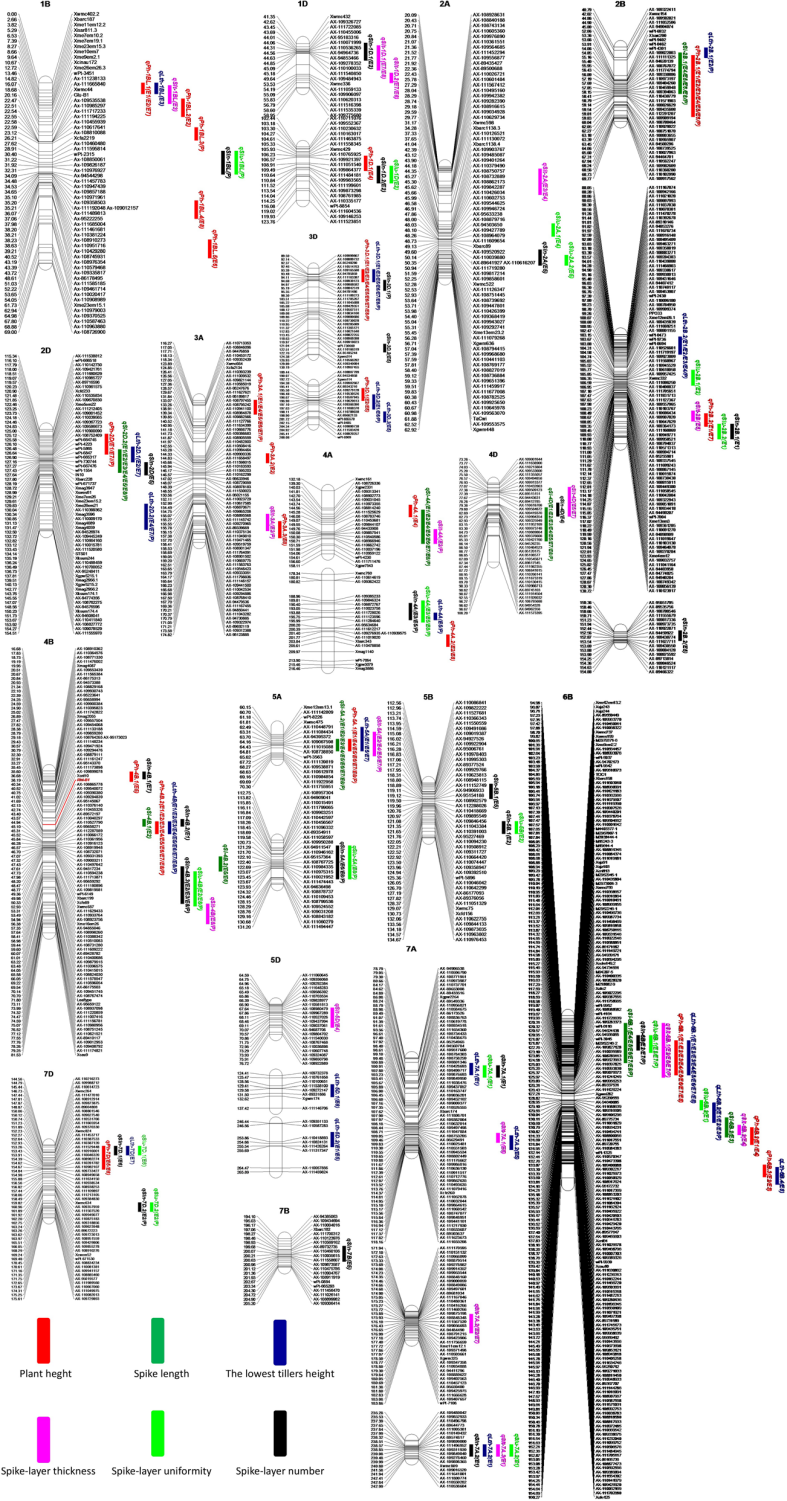
The location of QTL for wheat spike layer uniformity related traits based on a RIL population derived from Kenong 9204 and Jing 411. The short arms are at the top. The names of the marker loci and the QTL are listed to the right of the corresponding chromosomes. The positions of the marker loci are listed to the left of the corresponding chromosomes. The intervals of QTL were LOD > 2.0 with LOD peak values more than 2.5. Different colors of the QTL symbol indicate QTL for plant height, spike length, the lowest tillers height, spike layer thickness, spike layer uniformity and spike layer number. For more details, see QTL symbols at the left bottom of figure. Only the QTL for spike length that co-located with spike layer uniformity related traits were shown in this figure

A total of 99 putative additive QTL for the five SLURTs were detected in the nine data sets based on individual environment QTL mapping analysis. These QTL were distributed across all 21 wheat chromosomes except for 1A, 3B, 6A and 6D. Of these, 30, 40 and 29 QTL were mapped to the A, B and D genomes, respectively (Additional file 1: Table S10; Fig. 2). These QTL individually explained 1.05–39.62% of the phenotypic variance, with LOD values ranging from 2.00 to 34.01. Of these, 25 (25.3%) QTL for SLURTs were repeatedly detected in at least three of the nine data sets, and 11 (11.1%) were stable QTL that were verified in no less than five data sets (Additional file 1: Table S10; Table 3; Fig. 2). Combined QTL mapping analysis across environments verified 89 (89.9%) of the above-mentioned 99 QTL. In addition, 57 (57.6%) of the 99 QTL showed significant A by E interaction effects (Additional file 1: Table S11).

**Table 3:** Putative additive QTL for spike-layer uniformity related traits that were significant in no less than three of the nine data sets based on the KJ-RIL population

Trait^a^	QTL^b^	No. of data set^c^	LOD value^d^	PVE%^e^	Add effect^f^
PH	*qPh-1BL.1*	3	8.30	5.38	−2.55
	*qPh-2B.1*	7	5.79	4.59	2.09
	*qPh-2D*	3	5.03	3.34	1.69
	*qPh-3A.1*	6	4.59	3.33	1.75
	*qPh-3D.1*	9	10.45	8.53	2.69
	***qPh-4B.2***	9	23.56	**22.53**	−4.50
	*qPh-5A.1*	7	7.21	5.51	−2.21
	***qPh-6B.1***	9	12.79	**10.87**	−3.11
LTH	*qLth-2B.2*	5	4.19	4.33	1.88
	*qLth-2D.2*	3	8.39	4.07	1.67
	*qLth-3D.1*	7	4.98	5.87	2.07
	*qLth-3D.2*	3	3.09	2.95	−1.60
	***qLth-4B***	9	18.19	**25.04**	−4.37
	*qLth-5A*	3	3.22	3.48	−1.73
	***qLth-6B.1***	9	9.64	**12.68**	−3.10
	*qLth-6B.2*	4	4.50	4.13	−7.73
SLT	*qSlt-5A*	5	3.64	7.69	−1.33
	***qSlt-6B.1***	4	6.21	**13.71**	−1.86
SLN	*qSln-4A*	3	3.57	6.26	0.14
	*qSln-4B.3*	4	17.73	7.82	−0.15
	*qSln-5A*	3	2.82	5.56	−0.13
	***qSln-6B***	3	9.74	**18.67**	−0.21
SLU	*qSlu-4A*	4	2.84	6.40	−0.02
	*qSlu-4B*	3	4.46	8.00	0.01
	***qSlu-6B.1***	3	5.78	**12.04**	0.02

^a^LTH, The lowest tillers height; PH, Plant height; SL, Spike length; SLU, Spike-layer uniformity; SLN, Spike-layer number; SLT, Spike-layer thickness

^b^A putative major QTL is marked in bold typeface and is characterized by a mean LOD > 3.0 and a mean PVE > 10%, and a putative stable QTL is underlined when this locus was detected in at least five of the nine data sets

^c^The number of data sets where the corresponding QTL showed significance

^d^The average LOD value across data sets

^e^The average percentage of explained phenotypic variation by the QTL across data sets

^f^A positive sign indicates that the alleles from the Kenong9204 parent increased the corresponding trait value N, and a negative sign indicates that the alleles from the Jing411 parent increased the corresponding trait value

#### QTL for plant height

Twenty-three putative additive QTL for PH were identified in the nine data sets and were distributed on chromosomes 1BL (5 QTL), 1D, 2B (2 QTL), 2D, 3A (3 QTL), 3D (2 QTL), 4A (2 QTL), 4B (2 QTL), 5A, 6B (3 QTL) and 7D. These QTL individually explained 1.05–33.87% of the phenotypic variance with LOD values ranging from 2.03 to 34.01. Fourteen (60.9%) of the 23 QTL were verified in at least two of the nine data sets. Of these, *qPh-2B.1*, *qPh-3A.1*, *qPh-3D.1*, *qPh-4B.2*, *qPh-5A.1*, and *qPh-6B.1* were stable QTL that were identified repeatedly in no less than five data sets; *qPh-4B.2* and *qPh-6B.1* were major stable QTL for PH. All the 23 putative additive QTL for PH were significant in the combined QTL mapping analysis across environments; 15 (65.2%) of these QTL showed significant A by E interaction effects (Additional file 1: Table S11). Nine and 14 favorable alleles increasing PH were contributed by KN9204 and J411, respectively (Additional file 1: Table S10; Table 3; Fig. 2).

#### QTL for the lowest tillers height

Twenty putative additive QTL for LTH were mapped on chromosomes 1BL, 2B (2 QTL), 2D (2 QTL), 3D (3 QTL), 4A, 4B, 5A, 5D (2 QTL), 6B (3 QTL), 7A (3 QTL) and 7D. These QTL individually explained 1.73–39.62% of the phenotypic variance, with LOD values of 2.00–32.52. Twelve of the 20 QTL (60.0%) were verified in no less than two data sets, and four of these QTL, i.e., *qLth-2B.2*, *qLth-3D.1*, *qLth-4B* and *qLth-6B.1*, were stable across environments. *qLth-4B* and *qLth-6B.1* were major stable QTL that explained 25.04 and 12.68% of the phenotypic variance, respectively. All except *qLth-7A.2* were reproducibly identified in the combined QTL mapping analysis across environments; 8 (40.0%) of these QTL showed significant A by E interaction effects (Additional file 1: Table S11). Six and 14 QTL alleles increasing LTH were donated by KN9204 and J411, respectively (Additional file 1: Table S10; Table 3; Fig. 2).

#### QTL for spike-layer thickness

In total, 17 putative additive QTL for SLT were identified in the nine data sets and they were distributed on chromosomes 1BL, 1D (2 QTL), 2A, 2B, 3A, 4A, 4B, 4D (2 QTL), 5A, 5D, 6B (2 QTL), and 7A (3 QTL). These QTL individually accounted for 3.84–25.92% of the phenotypic variance with a LOD value of 2.08–11.01. Ten (58.8%) of the 17 QTL for SLT could be detected repeatedly in no less than two trials. *qSlt-5A* was the unique stable QTL for SLT that was verified in five of the nine data sets. In addition, *qSlt-6B.1* could be repeatedly detected in four data sets and explain 13.71% of the phenotypic variance with a LOD of 6.21. All the 17 QTL with the exception of *qSlt-1D.1*, *qSlt-6B.2* and *qSlt-7A.2* were verified in the combined QTL mapping analysis across environments; 8 (47.1%) of these QTL showed significant A by E interaction effects (Additional file 1: Table S11). Five and 12 QTL alleles increasing SLT originated from KN9204 and J411, respectively (Additional file 1: Table S10; Table 3; Fig. 2).

#### QTL for spike-layer number

Twenty-three QTL associated with SLN were detected in the nine data sets, which individually explained 4.03–36.52% of the phenotypic variation, with a LOD value of 2.01–19.24. These QTL were located on chromosomes 1BL, 1D (2 QTL), 2A, 2B (2 QTL), 2D, 3D (2 QTL), 4A, 4B (3 QTL), 4D, 5A, 5B (2 QTL), 6B, 7A (2 QTL), 7B and 7D (2 QTL). Only 5 (21.7%) of the 23 QTL for SLT could be reproducibly detected in no less than two trials. No stable QTL for SLN was identified herein. However, *qSln-4B.3* could be verified in four of the nine data sets, accounting for 7.82% of the phenotypic variance with a LOD value of 17.73. *qSln-6B* was reproducibly detected in three of the nine data sets, accounting for 18.67% of the phenotypic variance with a LOD value of 9.74 on average across environments. Combined QTL mapping analysis across environments verified 19 (82.6%) of the 23 QTL; 14 (60.9%) of these QTL showed significant A by E interaction effects (Additional file 1: Table S11). KN9204 and J411 contributed 12 and 11 QTL alleles for a higher SLN, respectively (Additional file 1: Table S10; Table 3).

#### QTL for spike-layer uniformity

For SLU, 16 QTL were mapped to chromosomes 1BL, 1D, 2A (2 QTL), 2B (2 QTL), 4A, 4B, 5A, 5B, 6B (2 QTL), 7A (2 QTL) and 7D (2 QTL). These QTL individually explained 5.04–18.43% of the phenotypic variation with a LOD value of 2.31–6.70. Only 5 (31.3%) of the 16 QTL for SLU could be verified in no less than two data sets, thus none of which was stable QTL. *qSlu-4A* could be identified in E3, E5, E8 and P, with a LOD vale of 2.84 and phenotypic variation explanation of 6.40% averagely across the four data sets. *qSlu-4B* was significant in E2, E8 and P, and individually explained 8.00% of the phenotypic variation with a LOD value of 4.46 on average. *qSlu-6B.1* was a major QTL that could be identified in E2, E7 and P, accounting for 12.04% of the phenotypic variation with a LOD value of 5.78 on average. All but *qSlu-7A.1* and *qSlu-7D.1* were reproducibly identified in the combined QTL mapping analysis across environments; 12 (75.0%) of these QTL showed significant A by E interaction effects (Additional file 1: Table S11). Nine and seven QTL alleles that increased SLU originated from KN9204 and J411, respectively (Additional file 1: Table S10; Table 3; Fig. 2).

## Discussion

### Genetic relationships between till height and spike-layer uniformity

SLU among inter-tillers have great effects on the population uniformity of wheat, thus influencing the marketability of the varieties. Genetic factors affecting wheat SLURTs have not been characterized before, though the QTL for plant type such as PH have been extensively reported previously [20–22]. Knowledge about the genetic relationships between till height and SLU is very limited. The present study showed that SLU had significant negative correlations with LTH and PH, albeit the absolute correlation coefficients were only 0.22 and 0.46, respectively (Table 1). These correlations indicated that the wheat plant with shorter PH and LTH tend to display more consistent spike vertical spatial distribution. Conversely, positive relationship between the LTH and SLU was found in rice [1], indicating that the genetic mechanisms controlling SLU in wheat and rice should be different.

Coincidence of QTL indicates either single QTL with pleiotropic effects or that the genomic region associated with these QTL harbors a cluster of linked genes associated with those traits. Four and six of the 16 putative additive QTL for SLU were co-located with the QTL for PH and LTH, respectively. This finding indicated that SLU and tiller height should be under different genetic control in most cases, which was consistent with the phenotypic correlation analysis (Table 1). In addition, Ma et al. [1] also found that no common QTL was shared with SLU and tiller height in rice.

Previous studies showed that the SLU among inter-tillers is mostly determined by genotypes [2–8]. The heritability of PH and LTH were more than twice as likely those of the three SLURTs (SLN, SLT and SLU) in this study (Additional file 1: Table S3). This finding confirmed the complex mode of SLURTs inheritance, indicating the difficulty in genetic improvement of SLU by direct selection in traditional wheat breeding programs. Therefore, it is of great necessity and importance to perform genetic analysis of SLU in wheat molecular breeding programs designed to obtain a desirable SLU among inter-tillers.

### Genetic relationships between spike-layer uniformity and yield-related traits

For crops with tillers such as wheat and rice, the characteristics SLU not only affect the appearance but also influence yield potential. Positive correlations were found between yield potential and SLU among 91 early and late hybrid rice cultivars [7, 8, 26]. Previous studies have shown that SLU affected yield potential through influencing some yield components such as TKW [7, 26, 27]. Knowledge about the genetic relationships between SLU and YRTs was very limited in wheat.

**Fig. 3:**
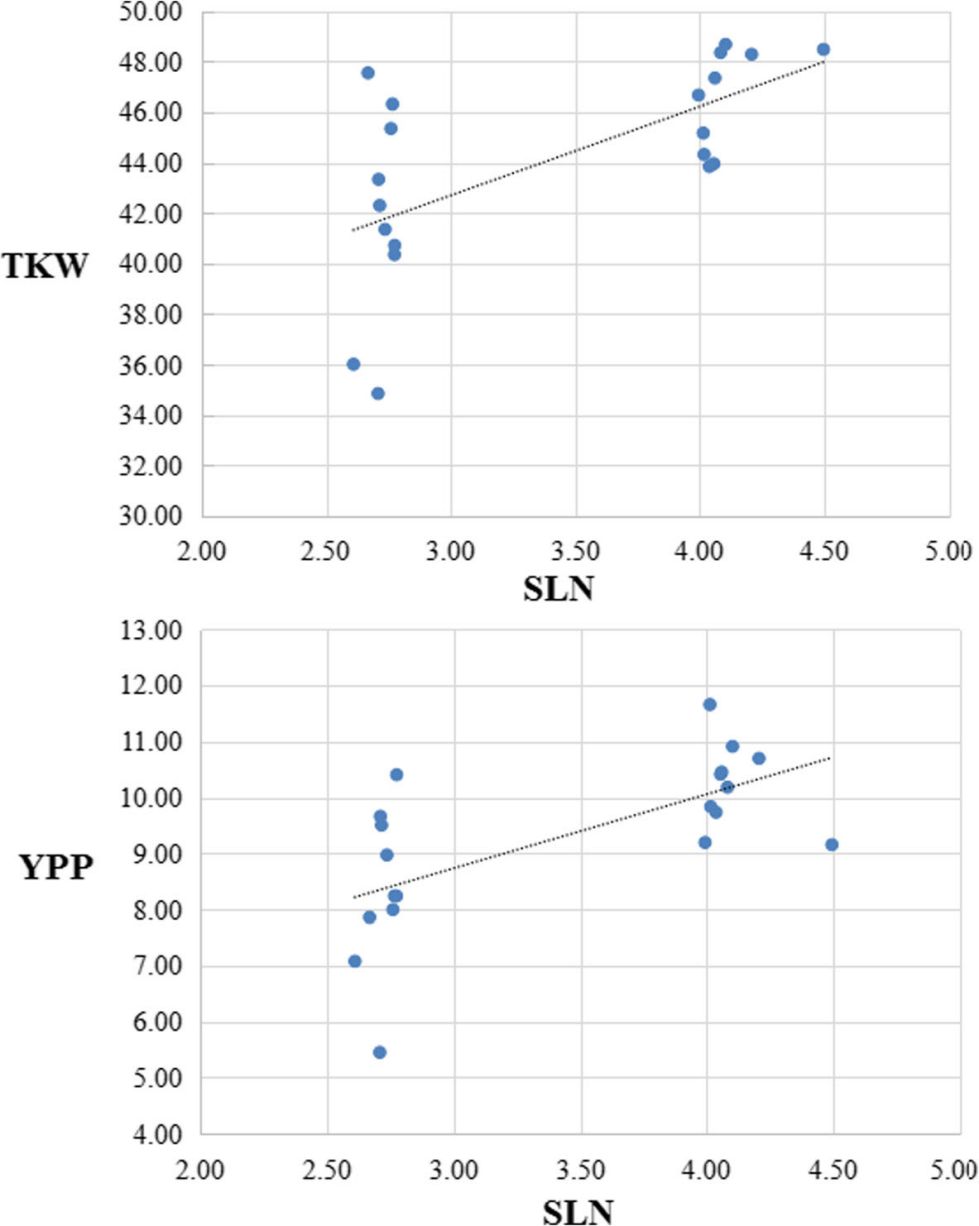
Comparing the effects of SLN on TKW and YPP using 20 RILs each ranked top-10 and ranked bottom-10 of the SLN based on regression analysis

**Fig. 4:**
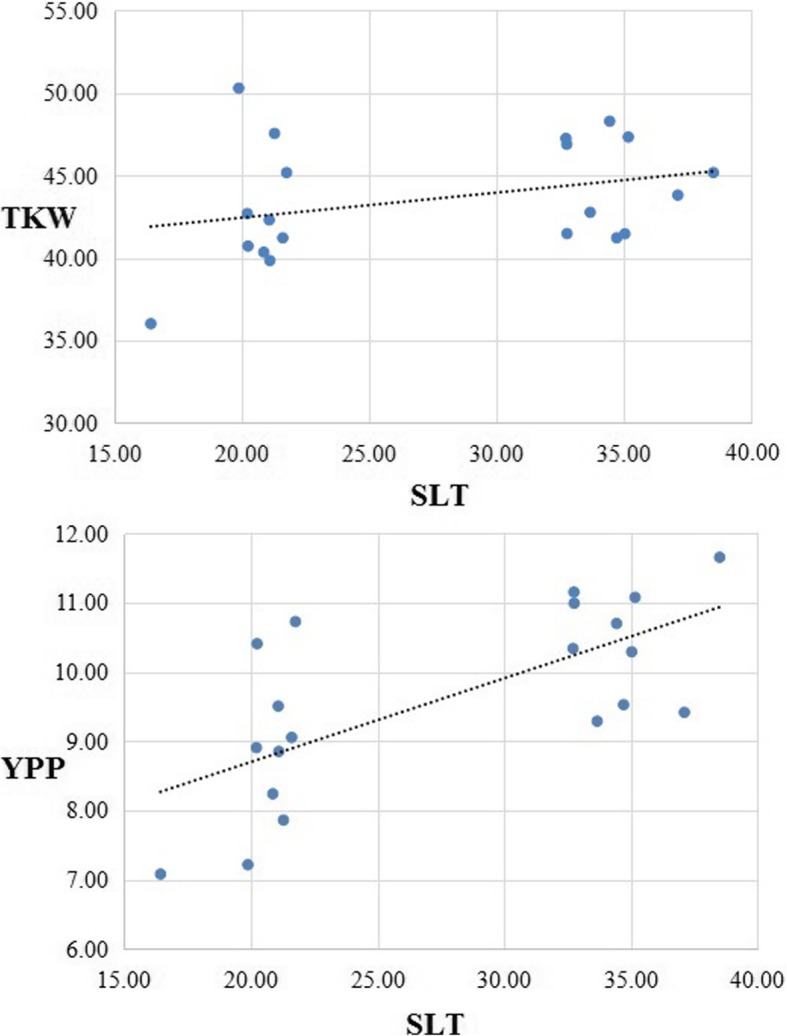
Comparing the effects of SLT on TKW and YPP using 20 RILs each ranked top-10 and ranked bottom-10 of the SLT based on regression analysis

Negative correlations were found between SLU and TKW and between SLU and YPP in wheat in this study (Table 2). SLU was found to have little effects on KNPS and SNPP. To more directly reflect the effects of SLN on TKW and YPP, 20 RILs each ranked top-10 and ranked bottom-10 of the SLN were sampled. Regression analysis showed that lines with more SLN tend to have larger kernels and yield more products (Fig. 3). Similar to SLN, lines with larger SLT were prone to have larger kernels and higher yield potential (Fig. 4). Yao [5] and Hu [6] have found that plants with uneven spike-layer to a certain extent are prone to have high yield potential. The above findings were inconsistent with that in rice, confirming that different genetic basis/network existed between wheat and rice in controlling YRTs through SLU [7, 8, 26]. In rice, lower photosynthesis rate of the tillers at the lower positions was attributed to a decrease in TKW and YPP [1]. This hypothesis/speculation may not work for wheat because that wheat spike is upright instead of floppy like rice. The photosynthetic rate of wheat spike might be increased if spikes have a desirable vertical spatial distribution instead of uniformity in an unique crowded horizontal space. All these hypothesis/speculation still need to be verified by more experiments in future. In addition, a canopy with uniform spike heights generally has insufficient number of spikes for high grain yield [1]. All these findings indicated that plants with uneven spike-layer to a certain extent should be an ideotype for super high-yield in wheat. Therefore, breeders might need to change traditional perceptions during the selection of desirable spike vertical spatial distribution in wheat breeding programs.

### QTL for SLT, SLN and SLU, and their potential use in wheat molecular breeding programs

In marker-assisted breeding the plant breeder takes advantage of the association between agronomic traits and molecular markers. QTL mapping analysis is an efficient method to identify markers linked with agronomic traits. Before a linkage marker can be used, the associations have to be assessed with a certain degree of accuracy and thus marker genotypes can be used as indicators of trait genotypes and phenotypes. If a QTL is independent of the environment, the implication is that its expression is stable regardless of differences in environment. A stable QTL consistent over environments is of great value for MAS in molecular breeding programs. In addition, close linkage molecular markers of a stable QTL should also be essential for their high efficient direct uses in MAS, which needs to narrow down a QTL to a quite small confidence interval.

QTL for SLT, SLN and SLU in wheat were first documented in the present study. Combined QTL mapping analysis across environments indicated that 61.0% of the identified QTL for the three SLURTs showed significant A by E interaction effects, consistent with their lower broad-sense heritability (Additional file 1: Table S3). Of these, *qSlt-5A* was the unique stable QTL for SLT that was verified in five of the nine data sets. In addition, *qSlu-4A*, *qSln-4B.3* and *qSlt-6B.1* could be repeatedly detected in four of the nine data sets. *qSlt-5A* was mapped to a 10 cM chromosomal interval covering 16 molecular markers; *qSlu-4A* was mapped to a 9 cM chromosomal interval covering 8 molecular markers; *qSln-4B.3* was mapped to a 15 cM chromosomal interval covering 18 molecular markers; and *qSlt-6B.1* was mapped to a 22 cM chromosomal interval covering 70 molecular markers. These results implied the difficulty in utilizing these QTL in MAS designed to improve wheat plant type due to their large confidence intervals as well as their instabilities across environments. Further fine mapping and map-based cloning of these QTL are essential for their further utilization in MAS.

## Conclusion

QTL for five SLURTs in multiple environments were first reported in this study. A total of 99 putative additive QTL for the five SLURTs were detected, 11 of which were stable across environments. Genetic relationships between SLURTs and yield indicated that plants with slight uneven spike-layer distribution should be an ideotype for super high-yield in wheat. The present study will provide assistance in understanding the genetic relationships between SLURTs and yield potential. The stable QTL for SLURTs identified herein may facilitate breeding new wheat varieties with scientifically reasonable spike-layer distribution by MAS.

## Additional files

Additional file 1: **Table S1.** Summary of the soil nitrate-nitrogen contents within the 0–20 cm layer in each environments. **Table S2.** Phenotypic performance of the two parental lines for spike-layer uniformity related traits in the eight environments; **Table S3.** Phenotypic performance for spike-layer uniformity related traits in the 188 KJ-RILs in eight environments; **Table S4.** Phenotypic correlation coefficients of the lowest tillers height (LTH) among the eight environments; **Table S5.** Phenotypic correlation coefficients of plant height (PH) among the eight environments; **Table S6.** Phenotypic correlation coefficients of spike length (SL) among the eight environments; **Table S7.** Phenotypic correlation coefficients of spike-layer thickness (SLT) among the eight environments; **Table S8.** Phenotypic correlation coefficients of spike-layer number (SLN) among the eight environments; **Table S9.** Phenotypic correlation coefficients of spike-layer uniformity (SLU) among the eight environments; **Table S10.** QTL with additive effects for spike layer uniformity related traits detected in the KJ-RIL population; **Table S11.** Combined QTL analysis across environments for spike layer uniformity related traits detected in the KJ-RIL population. (DOC 957 kb)

## Abbreviations

HN: High nitrogen treatment
J411: Jing411
KJ-RILs: Recombinant inbred line population derived from the cross between Kenong9204 and Jing411
KN9204: Kenong9204
KNPS: Kernel number per spike
LN: Low nitrogen treatment
LOD: Log-of-odds
LTH: The lowest tillers height
MAS: Marker-assisted selection
N: Nitrogen
PH: Plant height
QTL: Quantitative trait loci
RIL: Recombinant inbred line population
SL: Spike length
SLN: Spike-layer number
SLT: Spike-layer thickness
SLU: Spike-layer uniformity
SLURTs: Spike-layer uniformity related traits
SNPP: Spike number per plant
TKW: Thousand-kernel weight
YPP: Yield per plant
YRTs: Yield-related traits

## References

[1] Ma LY, Bao JS, Guo LB, Zeng DL, Li XM, Ji ZJ, Xia YW, Yang CD, Qian Q (2009). Quantitative trait loci for panicle layer uniformity identified in doubled haploid lines of rice in two environments. J Integr Plant Biol.

[2] Xu ZJ, Huang RD, Li HJ (2006). Difference and correlation of uniformity in rice population among varieties. J Shenyang Agric Univ.

[3] Zhang JG, Li C, Zhang SY, Shi YH, Yang GL, Zhao JS (1998). The comparison analysis for the uniformity of the population in different round grained rice varieties (I): the difference among different varieties of uniformity and the influence to the yield. J Jilin Agric Sci.

[4] Zhang JG, Li C, Zhang SY, Shi YH, Yang GL, Zhao JS (1999). The comparison and analysis of the uniformity of the population in different rounel_grained rice varieties (II): the influence of super sparse planting to variety shape and properties and uniformity. Jilin Agric Sci.

[5] Yao WC (2000). Studies on inheritance of spike layer uniformity of wheat. Seed.

[6] Hu YJ (2001). The difference of the spike-layer architecture and its relation to yield in winter wheat cultivars. Seed.

[7] Liu JF, Tang WB, Xiao YH, Yuan NS (2001). Relationship between plant evenness and yield characters of two-line hybrid late season rice. J Hunan Agric Univ (Nat Sci).

[8] Li JW, Yan QQ (2005). Studies on the relationship between the evenness degree and yield characters of hybrid early rice. Hunan Agric Sci.

[9] Wang WY, Xue MS (1987). Investigation and analysis of panicle layer uniformity in winter season wheat. Acta Agron Sin.

[10] Khush GS. Prospects of and approaches to increasing the genetic yield potential of rice. Rice Res in Asia, Progress and Priorities: CAB International and IRRI; 1996. p. 59–71.

[11] Peng S, Cassman KG, Virmani SS (1999). Yield potential trends of tropical rice since the release of IR8 and the challenge of increasing rice yield potential. Crop Sci.

[12] Peng S, Khush GS, Virk P, Tang Q, Zou Y (2008). Progress in ideotype breeding to increase rice yield potential. Field Crops Res.

[13] Jia J, Zhao S, Kong X, Li Y, Zhao G, He W, Appels R, Pfeifer M, Tao Y, Zhang X, Jing R, Zhang C, Ma Y, Gao L, Gao C, Spannagl M, Mayer KFX, Li D, Pan S, Zheng F, Hu Q, Xia X, Li J, Liang Q, Chen J, Wicker T, Gou C, Kuang H, He G, Luo Y, Keller B, Xia Q, Lu P, Wang J, Zou H, Zhang R, Xu J, Gao J, Middleton C, Quan Z, Liu G, Wang J, Yang H, Liu X, He Z, Mao L, Wang J, IWGSC (2013). The Aegilops tauschii draft genome sequence reveals a gene repertoire for wheat adaptation. Nature.

[14] Ling H, Zhao S, Liu D, Wang J, Sun H, Zhang C, Fan H, Li D, Dong L, Tao Y, Gao C, Wu H, Li Y, Cui Y, Guo X, Zheng S, Wang B, Yu K, Liang Q, Yang W, Lou X, Chen J, Feng M, Jian J, Zhang X, Luo G, Jiang Y, Liu J, Wang Z, Sha Y, Zhang B, Wu H, Tang D, Shen Q, Xue P, Zou S, Wang X, Liu X, Wang F, Yang Y, An X, Dong Z, Zhang K, Zhang X, Luo MC, Dvorak J, Tong Y, Wang J, Yang H, Li Z, Wang D, Zhang A, Wang J (2013). Draft genome of the wheat A-genome progenitor *Triticum urartu*. Nature.

[15] Choulet F, Alberti A, Theil S, Glover N, Barbe V, Daron J, Pingault L, Sourdille P, Couloux A, Paux E, Leroy P, Mangenot S, Guilhot N, Le Gouis J, Balfourier F, Alaux M, Jamilloux V, Poulain J, Durand C, Bellec A, Gaspin C, Safar J, Dolezel J, Rogers J, Vandepoele K, Aury JM, Mayer K, Berges H, Quesneville H, Wincker P, Feuillet C (2014). Structural and functional partitioning of bread wheat chromosome 3B. Science.

[16] Mayer KF, Rogers J, Doležel J, Pozniak C, Eversole K, Feuillet C, Gill B, Friebe B, Lukaszewski AJ, Sourdille P, Endo TR, Kubaláková M, Cíhalíková J, Dubská Z, Vrána J, Sperková R, Simková H, Febrer M, Clissold L, McLay K, Singh K, Chhuneja P, Singh NK, Khurana J, Akhunov E, Choulet F, Alberti A, Barbe V, Wincker P, Kanamori H, Kobayashi F, Itoh T, Matsumoto T, Sakai H, Tanaka T, Wu J, Ogihara Y, Handa H, Maclachlan PR, Sharpe A, Klassen D, Edwards D, Batley J, Olsen OA, Sandve SR, Lien S, Steuernagel B, Wulff B, Caccamo M, Ayling S, Ramirez-Gonzalez RH, Clavijo BJ, Wright J, Pfeifer M, Spannagl M, Martis MM, Mascher M, Chapman J, Poland JA, Scholz U, Barry K, Waugh R, Rokhsar DS, Muehlbauer GJ, Stein N, Gundlach H, Zytnicki M, Jamilloux V, Quesneville H, Wicker T, Faccioli P, Colaiacovo M, Stanca AM, Budak H, Cattivelli L, Glover N, Pingault L, Paux E, Sharma S, Appels R, Bellgard M, Chapman B, Nussbaumer T, Bader KC, Rimbert H, Wang S, Knox R, Kilian A, Alaux M, Alfama F, Couderc L, Guilhot N, Viseux C, Loaec M, Keller B, Praud S (2014). A chromosome-based draft sequence of the hexaploid bread wheat (*Triticum aestivum*) genome. Science.

[17] Zhao G, Zou C, Li K, Wang K, Li T, Gao L, Zhang X, Wang H, Yang Z, Liu X, Jiang W, Mao L, Kong X, Jiao Y, Jia J (2017). The Aegilops tauschii genome reveals multiple impacts of transposons. Nature Plants.

[18] Luo MC, Gu YQ, Puiu D, Wang H, Twardziok SO, Deal KR, Huo N, Zhu T, Wang L, Wang Y, McGuire PE, Liu S, Long H, Ramasamy RK, Rodriguez JC, Van SL, Yuan L, Wang Z, Xia Z, Xiao L, Anderson OD, Ouyang S, Liang Y, Zimin AV, Pertea G, Qi P, Bennetzen JL, Dai X, Dawson MW, Müller HG, Kugler K, Rivarola-Duarte L, Spannagl M, Mayer KFX, Lu FH, Bevan MW, Leroy P, Li P, You FM, Sun Q, Liu Z, Lyons E, Wicker T, Salzberg SL, Devos KM, Dvořák J (2017). Genome sequence of the progenitor of the wheat D genome *Aegilops tauschii*. Nature.

[19] Ling HQ, Ma B, Shi X, Liu H, Dong L, Sun H, Cao Y, Gao Q, Zheng S, Li Y, Yu Y, Du H, Qi M, Li Y, Lu H, H Y, Cui Y, Wang N, Chen C, Wu H, Zhao Y, Zhang J, Li Y, Zhou W, Zhang B, Hu W, van Eijk MJT, Tang J, Witsenboer HMA, Zhao S, Li Z, Zhang A, Wang D, Liang C (2018). Genome sequence of the progenitor of wheat a subgenome *Triticum urartu*. Nature.

[20] Cui F, Li J, Ding A, Zhao C, Wang L, Wang X, Li S, Bao Y, Li X, Feng D, Kong L, Wang H (2011). Conditional QTL mapping for plant height with respect to the length of the spike and internode in two mapping populations of wheat. Theor Appl Genet.

[21] Bai C, Liang Y, Hawkesford MJ (2013). Identification of QTL associated with seedling root traits and their correlation with plant height in wheat. J Exp Bot.

[22] Zhang N, Fan XL, Cui F, Zhao CH, Zhang W, Zhao XQ, Yang LJ, Pan RQ, Chen M, Han J, Ji J, Liu DC, Zhao ZW, Tong YP, Zhang AM, Wang T, Li JM (2017). Characterization of the temporal and spatial expression of wheat (*Triticum aestivum* L.) plant height at the QTL level and their influence on yield related traits. Theor Appl Genet.

[23] Cui F, Zhang N, Fan XL, Zhang W, Zhao CH, Yang LJ, Pan RQ, Chen M, Han J, Zhao XQ, Ji J, Tong YP, Zhang HX, Jia JZ, Zhao GY, Li JM (2017). Utilization of a Wheat660K SNP array-derived high-density genetic map for high-resolution mapping of a major QTL for kernel number. Sci Reports.

[24] Cui F, Fan XL, Chen M, Zhang N, Zhao CH, Zhang W, Han J, Ji J, Zhao XQ, Yang LJ, Zhao ZW, Tong YP, Wang T, Li JM (2016). QTL detection for wheat kernel size and quality and the responses of these traits to low nitrogen stress. Theor Appl Genet.

[25] Fan XL, Cui F, Zhao CH, Zhang W, Yang LJ, Zhao XQ, Han J, Su QN, Ji J, Zhao ZW, Tong YP, Li JM. QTL for flag leaf size and their influence on yield-related traits in wheat (*Triticum aestivum* L.). Mol Breeding. 2015;35.

[26] Zhao DH, Yan QQ, Wang JR, Wan HQ (2005). Study on relationship between population uniformity and yield characters of late hybrid rice. Guangxi Agric Sci.

[27] Zhang WW, Zhu DF, Zhang YP, Chen HZ, Lin XQ (2004). Analysis of panicle uniformity and yield of rice hybrid in planting density. Southwest China J Agric Sci.

