# Supplementary material for: QTL for spike-layer uniformity and their influence on yield-related traits in wheat
Source: BMC Genet. 2019 Feb 28;20:23. doi: 10.1186/s12863-019-0730-3 (PMC6396499; doi:10.1186/s12863-019-0730-3)
Supplement: Supplementary file 1 — Table S1. Summary of the soil nitrate-nitrogen contents within the 0–20 cm layer in each environments. Table S2. Phenotypic performance of the two parental lines for spike-layer uniformity related traits in the eight environments; Table S3. Phenotypic performance for spike-layer uniformity related traits in the 188 KJ-RILs in eight environments; Table S4. Phenotypic correlation coefficients of the lowest tillers height (LTH) among the eight environments; Table S5. Phenotypic correlation coefficients of plant height (PH) among the eight environments; Table S6. Phenotypic correlation coefficients of spike length (SL) among the eight environments; Table S7. Phenotypic correlation coefficients of spike-layer thickness (SLT) among the eight environments; Table S8. Phenotypic correlation coefficients of spike-layer number (SLN) among the eight environments; Table S9. Phenotypic correlation coefficients of spike-layer uniformity (SLU) among the eight environments; Table S10. QTL with additive effects for spike layer uniformity related traits detected in the KJ-RIL population; Table S11. Combined QTL analysis across environments for spike layer uniformity related traits detected in the KJ-RIL population. (DOC 957 kb) [file 12863_2019_730_MOESM1_ESM.doc]

Supplementary Data for the manuscript entitled,

**QTL for spike-layer uniformity and their influence on yield-related traits in wheat**

Chunhua Zhao^1†^, Na Zhang^2†^, Yongzhen Wu^1†^, Han Sun^1^, Cheng Liu^3^, Xiaoli Fan^4*^, Xuemei Yan^5^, Hongxin Xu^6^, Jun Ji^2*^ and Fa Cui^1*^

**Supplementary Tables**

**Table S1 Summary of the soil nitrate-nitrogen contents within the 0–20 cm layer in each environments…………………………………………………………………………………………………………..2**

**Table S2 Phenotypic performance of the two parental lines for spike-layer uniformity related traits in the eight environments………………………………………………………………………………………………………………..3**

**Table S3 Phenotypic performance for spike-layer uniformity related traits in the 188 KJ-RILs in eight environments…………………………………………………………………………………………………………..……4**

**Table S4 Phenotypic correlation coefficients of the lowest tillers height (LTH) among the eight environments………………………………………………….……………………….……………………….…...………6**

**Table S5 Phenotypic correlation coefficients of plant height (PH) among the eight environments……………...……7**

**Table S6 Phenotypic correlation coefficients of spike length (SL) among the eight environments……………..…….8**

**Table S7 Phenotypic correlation coefficients of spike-layer thickness (SLT) among the eight environments…….….9**

**Table S8 Phenotypic correlation coefficients of spike-layer number (SLN) among the eight environments….…..10**

**Table S9 Phenotypic correlation coefficients of spike-layer uniformity (SLU) among the eight environments…...11**

**Table S10** **QTL with additive effects for spike layer uniformity related traits detected in the KJ-RIL population...12**

**Table S11 Combined QTL analysis across environments for spike layer uniformity related traits detected in the KJ-RIL population………………………………………….……………………….……………………….…...………18**

**Table S1 Summary of the soil nitrate-nitrogen contents within the 0–20 cm layer in each environments**

| Environments | Year | Location | Soli nitrate-N contents (mg kg^-1^) | Soli total-N contents (mg kg^-1^) |
| --- | --- | --- | --- | --- |
| E1 | 2013–2014 | Shijiazhuang | 39.6 | 126.2 |
| E2 | 2013–2014 | Shijiazhuang | 41.2 | 128.3 |
| E3 | 2013–2014 | Shijiazhuang | 28.4 | 103.1 |
| E4 | 2014–2015 | Shijiazhuang | 27.8 | 102.6 |
| E5 | 2016–2017 | Yantai | 49.2 | 135.6 |
| E6 | 2017–2018 | Yantai | 55.2 | 137.9 |
| E7 | 2016–2017 | Yantai | 46.8 | 133.6 |
| E8 | 2017–2018 | Yantai | 51.3 | 130.2 |

**Table S2 Phenotypic performance of the two parental lines for spike-layer uniformity related traits in the eight environments**

| Parents^a^ | Traits^b^ | Environments^c^ | | | | | | | |
| --- | --- | --- | --- | --- | --- | --- | --- | --- | --- |
|  |  | E1 | E2 | E3 | E4 | E5 | E6 | E7 | E8 |
| KN9204 | LTH (cm) | 55.8 | 54.9 | 43.3 | 44.5 | 53.6 | 52.5 | 43.4 | 43.9 |
|  | PH (cm) | 73.6 | 75.4 | 68.3 | 70.0 | 75.2 | 74.2 | 67.9 | 70.1 |
|  | SL (cm) | 7.7 | 7.7 | 7.1 | 7.6 | 8.6 | 8.1 | 8.1 | 8.3 |
|  | SLN | 3.3 | 3.7 | 4.5 | 4.4 | 3.5 | 3.7 | 4.0 | 4.2 |
|  | SLT (cm) | 25.5 | 28.2 | 32.1 | 33.1 | 30.2 | 29.8 | 32.6 | 34.5 |
|  | SLU | 0.30 | 0.27 | 0.22 | 0.23 | 0.28 | 0.27 | 0.25 | 0.24 |
| J411 | LTH (cm) | 72.1 | 75.4 | 58.6 | 52.0 | 72.0 | 71.0 | 57.2 | 59.4 |
|  | PH (cm) | 88.6 | 92.0 | 76.7 | 75.2 | 89.4 | 87.9 | 80.3 | 79.8 |
|  | SL (cm) | 8.1 | 8.2 | 7.9 | 8.1 | 9.1 | 8.9 | 8.8 | 8.6 |
|  | SLN | 3.0 | 3.0 | 3.3 | 3.9 | 2.9 | 2.9 | 3.6 | 3.4 |
|  | SLT (cm) | 24.6 | 24.8 | 26.0 | 31.3 | 26.5 | 25.8 | 31.9 | 29.0 |
|  | SLU | 0.33 | 0.33 | 0.30 | 0.26 | 0.34 | 0.34 | 0.28 | 0.30 |

^a^ KN9204, Kenong 9204; J411, Jing411

^b^ LTH, The lowest tillers height in centimeter; PH, Plant height in centimeter; SL, Spike length in centimeter; SLU, Spike-layer uniformity; SLN, Spike-layer number; SLT, Spike-layer thickness centimeter.

^c^E1, E2, E3, E4, E5, E6, E7 and E8 represent the environments of 2013–2014 in Shijiazhuang with normal nitrogen treatment, 2014–2015 in Shijiazhuang with normal nitrogen treatment, 2013–2014 in Shijiazhuang with low nitrogen treatment, 2014–2015 in Shijiazhuang with low nitrogen treatment, 2016–2017 in Yantai with normal nitrogen treatment, 2017–2018 in Yantai with normal nitrogen treatment, 2016–2017 in Yantai with low nitrogen treatment, 2017–2018 in Yantai with low nitrogen treatment, respectively. *P* presents the average value of the corresponding traits across the 8 environments

**Table S3** **Phenotypic performance for spike-layer uniformity related traits in the 188 KJ-RILs in eight environments**

| **Trait (*h^2^*)^a^** | **Env.^b^** | **Mean** | **Variance** | **Std.** | **Skewness** | **Kurtosis** | **Minimum** | **Maximum** | **Range** | **W–test^c^** | ***P*–value^d^** |
| --- | --- | --- | --- | --- | --- | --- | --- | --- | --- | --- | --- |
| LTH (cm)  (64.8%) | E1 | 65.78 | 116.85 | 10.81 | 0.08 | –0.54 | 41.40 | 91.80 | 50.40 | 0.97 | 0.04 |
|  | E2 | 65.46 | 109.83 | 10.48 | 0.30 | –0.47 | 44.20 | 95.80 | 51.60 | 0.97 | 0.01 |
|  | E3 | 54.66 | 65.18 | 8.07 | –0.21 | 0.02 | 29.30 | 75.30 | 46.00 | 0.99 | **0.80** |
|  | E4 | 53.04 | 73.13 | 8.55 | –0.03 | 0.04 | 25.20 | 75.80 | 50.60 | 0.99 | **0.92** |
|  | E5 | 59.12 | 90.95 | 9.54 | 0.49 | 0.66 | 38.56 | 93.60 | 55.04 | 0.98 | **0.12** |
|  | E6 | 59.43 | 88.09 | 9.39 | 0.13 | –0.25 | 37.40 | 83.10 | 45.70 | 0.98 | **0.19** |
|  | E7 | 59.45 | 59.81 | 7.73 | 0.23 | 0.20 | 39.50 | 83.60 | 44.10 | 0.99 | **0.72** |
|  | E8 | 58.83 | 69.28 | 8.32 | 0.23 | 0.07 | 40.30 | 85.40 | 45.10 | 0.98 | **0.30** |
|  | P | 59.50 | 58.69 | 7.66 | 0.10 | –0.04 | 39.74 | 84.70 | 44.96 | 0.99 | **0.90** |
| PH (cm)  (85.5%) | E1 | 84.31 | 126.31 | 11.24 | 0.06 | –0.68 | 55.60 | 111.00 | 55.40 | 0.97 | **0.07** |
|  | E2 | 85.07 | 130.62 | 11.43 | –0.01 | –0.59 | 54.70 | 114.40 | 59.70 | 0.98 | **0.24** |
|  | E3 | 73.56 | 62.64 | 7.91 | 0.02 | 0.03 | 46.20 | 94.40 | 48.20 | 0.99 | **0.75** |
|  | E4 | 72.22 | 60.30 | 7.77 | –0.02 | 0.15 | 45.80 | 95.00 | 49.20 | 0.99 | **0.95** |
|  | E5 | 81.10 | 113.08 | 10.63 | 0.33 | 0.11 | 54.20 | 112.40 | 58.20 | 0.98 | **0.20** |
|  | E6 | 81.11 | 109.15 | 10.45 | 0.24 | 0.16 | 53.90 | 112.20 | 58.30 | 0.98 | **0.45** |
|  | E7 | 75.60 | 81.47 | 9.03 | 0.30 | 0.13 | 48.90 | 102.40 | 53.50 | 0.98 | **0.53** |
|  | E8 | 74.46 | 77.59 | 8.81 | 0.18 | –0.01 | 47.20 | 98.80 | 51.60 | 0.99 | **0.70** |
|  | P | 78.47 | 83.35 | 9.13 | 0.10 | –0.15 | 50.81 | 104.06 | 53.24 | 0.99 | **0.82** |
| SL (cm)  (67.8%) | E1 | 7.76 | 0.62 | 0.79 | 0.38 | 0.30 | 5.60 | 10.20 | 4.60 | 0.98 | **0.21** |
|  | E2 | 7.75 | 0.57 | 0.76 | 0.32 | 0.76 | 5.60 | 10.30 | 4.70 | 0.98 | **0.60** |
|  | E3 | 7.41 | 0.60 | 0.77 | 0.36 | 0.71 | 5.40 | 10.10 | 4.70 | 0.98 | **0.50** |
|  | E4 | 7.52 | 0.59 | 0.77 | 0.23 | 0.64 | 5.30 | 10.50 | 5.20 | 0.99 | **0.99** |
|  | E5 | 8.81 | 0.75 | 0.87 | –0.13 | 0.04 | 6.30 | 10.82 | 4.52 | 0.98 | **0.43** |
|  | E6 | 8.78 | 0.68 | 0.83 | 0.10 | –0.07 | 6.40 | 11.00 | 4.60 | 0.99 | **0.77** |
|  | E7 | 8.58 | 0.75 | 0.87 | –0.07 | 1.58 | 5.32 | 11.30 | 5.98 | 0.98 | **0.69** |
|  | E8 | 8.60 | 0.72 | 0.85 | –0.19 | 0.55 | 5.32 | 10.90 | 5.58 | 0.99 | **0.95** |
|  | P | 8.14 | 0.47 | 0.69 | 0.19 | 0.39 | 6.30 | 10.12 | 3.82 | 0.98 | **0.44** |
| SLN  (23.5%) | E1 | 3.39 | 0.35 | 0.59 | 0.10 | 0.15 | 1.96 | 5.17 | 3.21 | 0.98 | **0.52** |
|  | E2 | 3.54 | 0.28 | 0.52 | 0.42 | 0.80 | 2.05 | 5.31 | 3.26 | 0.98 | **0.27** |
|  | E3 | 3.54 | 0.42 | 0.65 | 0.85 | 0.81 | 2.41 | 5.94 | 3.53 | 0.95 | 0.00 |
|  | E4 | 3.53 | 0.46 | 0.68 | 0.18 | 0.22 | 1.55 | 5.34 | 3.79 | 0.98 | **0.40** |
|  | E5 | 3.50 | 0.46 | 0.68 | 0.25 | 0.33 | 1.83 | 5.68 | 3.85 | 0.98 | **0.62** |
|  | E6 | 3.47 | 0.43 | 0.65 | 0.23 | 0.10 | 1.91 | 5.35 | 3.44 | 0.98 | **0.13** |
|  | E7 | 2.79 | 0.36 | 0.60 | 0.08 | –0.50 | 1.45 | 4.58 | 3.13 | 0.98 | **0.36** |
|  | E8 | 2.78 | 0.40 | 0.64 | 0.38 | –0.27 | 1.61 | 4.97 | 3.36 | 0.97 | 0.01 |
|  | P | 3.34 | 0.13 | 0.36 | 0.43 | –0.17 | 2.61 | 4.50 | 1.89 | 0.97 | 0.03 |
| SLT (cm)  (30.7%) | E1 | 26.27 | 26.31 | 5.13 | 0.46 | 0.65 | 14.30 | 45.60 | 31.30 | 0.98 | **0.35** |
|  | E2 | 27.38 | 19.37 | 4.40 | 0.30 | 0.97 | 15.00 | 41.40 | 26.40 | 0.97 | **0.10** |
|  | E3 | 26.34 | 29.10 | 5.39 | 0.65 | 0.60 | 14.40 | 45.40 | 31.00 | 0.97 | 0.01 |
|  | E4 | 26.68 | 33.68 | 5.80 | 0.20 | 0.18 | 11.30 | 42.20 | 30.90 | 0.98 | **0.17** |
|  | E5 | 30.47 | 50.10 | 7.08 | –0.55 | **2.94** | 0.00 | 49.94 | 49.94 | 0.96 | 0.00 |
|  | E6 | 29.81 | 60.55 | 7.78 | –0.92 | **3.02** | 0.00 | 49.74 | 49.74 | 0.93 | 0.00 |
|  | E7 | 23.99 | 33.37 | 5.78 | 0.18 | –0.06 | 10.70 | 40.54 | 29.84 | 0.98 | **0.34** |
|  | E8 | 23.61 | 40.68 | 6.38 | –0.23 | **1.10** | 0.00 | 40.30 | 40.30 | 0.97 | **0.08** |
|  | P | 27.11 | 11.21 | 3.35 | 0.27 | 0.73 | 16.44 | 38.51 | 22.07 | 0.99 | **0.95** |
| SLU  (21.0%) | E1 | 0.30 | 0.00 | 0.06 | 0.97 | **1.09** | 0.19 | 0.51 | 0.32 | 0.94 | 0.00 |
|  | E2 | 0.29 | 0.00 | 0.04 | 0.96 | **3.08** | 0.19 | 0.49 | 0.30 | 0.95 | 0.00 |
|  | E3 | 0.29 | 0.00 | 0.05 | 0.07 | –0.37 | 0.17 | 0.42 | 0.25 | 0.98 | **0.26** |
|  | E4 | 0.29 | 0.00 | 0.06 | **1.66** | **5.74** | 0.19 | 0.65 | 0.46 | 0.90 | 0.00 |
|  | E5 | 0.30 | 0.00 | 0.06 | **1.07** | **1.73** | 0.18 | 0.55 | 0.37 | 0.94 | 0.00 |
|  | E6 | 0.30 | 0.00 | 0.06 | 0.91 | **1.04** | 0.19 | 0.52 | 0.34 | 0.94 | 0.00 |
|  | E7 | 0.38 | 0.01 | 0.09 | 0.96 | 0.80 | 0.22 | 0.69 | 0.47 | 0.93 | 0.00 |
|  | E8 | 0.38 | 0.01 | 0.09 | 0.50 | –0.50 | 0.20 | 0.62 | 0.42 | 0.95 | 0.00 |
|  | P | 0.30 | 0.00 | 0.03 | 0.07 | –0.42 | 0.22 | 0.38 | 0.16 | 0.98 | **0.31** |

**^a^** LTH, The lowest tillers height; PH, Plant height; SL, Spike length; SLU, Spike-layer uniformity; SLN, Spike-layer number; SLT, Spike-layer thickness. *H^2^*: Arabic numerals in parentheses are estimated broad-sense heritabilities of the corresponding traits.

**^b^** E1, E2, E3, E4, E5, E6, E7 and E8 represent the environments of 2013–2014 in Shijiazhuang with normal nitrogen treatment, 2014–2015 in Shijiazhuang with normal nitrogen treatment, 2013–2014 in Shijiazhuang with low nitrogen treatment, 2014–2015 in Shijiazhuang with low nitrogen treatment, 2016–2017 in Yantai with normal nitrogen treatment, 2017–2018 in Yantai with normal nitrogen treatment, 2016–2017 in Yantai with low nitrogen treatment, 2017–2018 in Yantai with low nitrogen treatment, respectively. *P* presents the average value of the corresponding traits across the 8 environments

**^c^** *W*-test: The Shapiro Wilk W-statistic for the test of normality.

**^d^** *P*-Value: *P*-value of the W-test of normality. *P*>0.05, the corresponding traits follow a normality distribution in the 188 KJ-RILs.

**Table S4 Phenotypic correlation coefficients of the lowest tillers height (LTH) among the eight environments**

|  | LTH-E1 | LTH-E2 | LTH-E3 | LTH-E4 | LTH-E5 | LTH-E6 | LTH-E7 | LTH-E8 |
| --- | --- | --- | --- | --- | --- | --- | --- | --- |
| LTH-E1 | 1 |  |  |  |  |  |  |  |
| LTH-E2 | 0.864** | 1 |  |  |  |  |  |  |
| LTH-E3 | 0.668** | 0.639** | 1 |  |  |  |  |  |
| LTH-E4 | 0.622** | 0.618** | 0.701** | 1 |  |  |  |  |
| LTH-E5 | 0.657** | 0.672** | 0.510** | 0.492** | 1 |  |  |  |
| LTH-E6 | 0.700** | 0.719** | 0.545** | 0.552** | 0.696** | 1 |  |  |
| LTH-E7 | 0.654** | 0.676** | 0.678** | 0.598** | 0.665** | 0.673** | 1 |  |
| LTH-E8 | 0.484** | 0.489** | 0.471** | 0.483** | 0.447** | 0.473** | 0.541** | 1 |

^**^ represents that correlation is significant at when *P* < 0.01 level.

**Table S5** Phenotypic correlation coefficients of plant height (PH) among the eight environments

|  | PH-E1 | PH-E2 | PH-E3 | PH-E4 | PH-E5 | PH-E6 | PH-E7 | PH-E8 |
| --- | --- | --- | --- | --- | --- | --- | --- | --- |
| PH-E1 | 1 |  |  |  |  |  |  |  |
| PH-E2 | 0.959** | 1 |  |  |  |  |  |  |
| PH-E3 | 0.852** | 0.864** | 1 |  |  |  |  |  |
| PH-E4 | 0.862** | 0.857** | 0.905** | 1 |  |  |  |  |
| PH-E5 | 0.881** | 0.881** | 0.807** | 0.830** | 1 |  |  |  |
| PH-E6 | 0.884** | 0.885** | 0.824** | 0.830** | 0.897** | 1 |  |  |
| PH-E7 | 0.893** | 0.890** | 0.863** | 0.873** | 0.868** | 0.897** | 1 |  |
| PH-E8 | 0.869** | 0.871** | 0.876** | 0.883** | 0.824** | 0.869** | 0.920** | 1 |

^**^ represents that correlation is significant at when *P* < 0.01 level.

**Table S6 Phenotypic correlation coefficients of spike length (SL) among the eight environments**

|  | SL-E1 | SL-E2 | SL-E3 | SL-E4 | SL-E5 | SL-E6 | SL-E7 | SL-E8 |
| --- | --- | --- | --- | --- | --- | --- | --- | --- |
| SL-E1 | 1 |  |  |  |  |  |  |  |
| SL-E2 | 0.780** | 1 |  |  |  |  |  |  |
| SL-E3 | 0.700** | 0.675** | 1 |  |  |  |  |  |
| SL-E4 | 0.719** | 0.719** | 0.779** | 1 |  |  |  |  |
| SL-E5 | 0.733** | 0.727** | 0.585** | 0.716** | 1 |  |  |  |
| SL-E6 | 0.695** | 0.736** | 0.597** | 0.685** | 0.757** | 1 |  |  |
| SL-E7 | 0.608** | 0.640** | 0.511** | 0.617** | 0.673** | 0.654** | 1 |  |
| SL-E8 | 0.630** | 0.706** | 0.586** | 0.675** | 0.667** | 0.699** | 0.822** | 1 |

^**^ represents that correlation is significant at when *P* < 0.01 level.

**Table S7 Phenotypic correlation coefficients of spike-layer thickness (SLT) among the eight environments**

|  | SLT-E1 | SLT-E2 | SLT-E3 | SLT-E4 | SLT-E5 | SLT-E6 | SLT-E7 | SLT-E8 |
| --- | --- | --- | --- | --- | --- | --- | --- | --- |
| SLT-E1 | 1 |  |  |  |  |  |  |  |
| SLT-E2 | 0.440** | 1 |  |  |  |  |  |  |
| SLT-E3 | 0.284** | 0.319** | 1 |  |  |  |  |  |
| SLT-E4 | 0.135 | 0.266** | 0.337** | 1 |  |  |  |  |
| SLT-E5 | 0.230** | 0.157* | 0.239** | -0.003 | 1 |  |  |  |
| SLT-E6 | 00.118 | 0.328** | 0.214** | 0.155* | 0.334** | 1 |  |  |
| SLT-E7 | 0.208** | 0.283** | 0.248** | 00.143 | 0.335** | 0.309** | 1 |  |
| SLT-E8 | 0.358** | 0.232** | 0.223** | 0.186* | 0.221** | 0.218** | 0.369** | 1 |

^**^ represents that correlation is significant at when *P* < 0.01 level; ^*^ represents that correlation is significant at when *P* < 0.05 level.

**Table S8 Phenotypic correlation coefficients of spike-layer number (SLN) among the eight environments**

|  | SLN-E1 | SLN-E2 | SLN-E3 | SLN-E4 | SLN-E5 | SLN-E6 | SLN-E7 | SLN-E8 |
| --- | --- | --- | --- | --- | --- | --- | --- | --- |
| SLN-E1 | 1 |  |  |  |  |  |  |  |
| SLN-E2 | 0.340** | 1 |  |  |  |  |  |  |
| SLN-E3 | 0.268** | 0.324** | 1 |  |  |  |  |  |
| SLN-E4 | 0.070 | 0.222** | 0.371** | 1 |  |  |  |  |
| SLN-E5 | 0.187* | 0.191** | 0.205** | -0.110 | 1 |  |  |  |
| SLN-E6 | 0.149* | 0.302** | 00.112 | 0.051 | 0.192** | 1 |  |  |
| SLN-E7 | 0.138 | 0.191* | 0.242** | 0.132 | 0.203* | 0.271** | 1 |  |
| SLN-E8 | 0.333** | 0.216** | 0.267** | 0.169* | 0.200** | 0.177* | 0.369** | 1 |

^**^ represents that correlation is significant at when *P* < 0.01 level; ^*^ represents that correlation is significant at when *P* < 0.05 level.

**Table S9 Phenotypic correlation coefficients of spike-layer uniformity (SLU) among the eight environments**

|  | SLU-E1 | SLU-E2 | SLU-E3 | SLU-E4 | SLU-E5 | SLU-E6 | SLU-E7 | SLU-E8 |
| --- | --- | --- | --- | --- | --- | --- | --- | --- |
| SLU-E1 | 1 |  |  |  |  |  |  |  |
| SLU-E2 | 0.266** | 1 |  |  |  |  |  |  |
| SLU-E3 | 0.255** | 0.294** | 1 |  |  |  |  |  |
| SLU-E4 | 0.036 | 0.180* | 0.318** | 1 |  |  |  |  |
| SLU-E5 | 0.170* | 0.153* | 0.229** | -0.065 | 1 |  |  |  |
| SLU-E6 | 0.155* | 0.325** | 00.147 | 0.009 | 0.118 | 1 | 0. |  |
| SLU-E7 | 0.118 | 0.166* | 0.243** | 0.059 | 0.227** | 0.272** | 1 |  |
| SLU-E8 | 0.321** | 0.233** | 0.273** | 0.11 | 0.189* | 0.094 | 0.344** | 1 |

^**^ represents that correlation is significant at when *P* < 0.01 level; ^*^ represents that correlation is significant at when *P* < 0.05 level.

**Table S10 QTL with additive effects for spike layer uniformity related traits detected in the KJ-RIL population**

| Trait | QTL | Environment | Position | Flanking Markers | **LOD value** | **PVE (%)** | Add effect |
| --- | --- | --- | --- | --- | --- | --- | --- |
| PH | *qPh-1BL.1* | E1 | 2.60 | *Xwmc402.2−Xbarc187* | **4.08** | 2.29 | −1.80 |
|  |  | E2 | 2.80 | *Xbarc187−Xme11em12.2* | **14.65** | **10.06** | −3.85 |
|  |  | E7 | 2.60 | *Xwmc402.2−Xbarc187* | **6.37** | 4.40 | −2.00 |
|  | *qPh-1BL.2* | E2 | 9.60 | *Xme10em7−Xme9em2.1* | **8.15** | 4.84 | 2.62 |
|  | *qPh-1BL.3* | P | 20.80 | *Glu-B1−Ax-109535538* | **4.09** | 2.94 | −1.58 |
|  | *qPh-1BL.4* | E8 | 49.20 | *Ax-86178495−Ax-111585185* | **4.43** | 4.57 | −1.88 |
|  | *qPh-1BL.5* | E6 | 64.20 | *Ax-110979003−Ax-109370525* | **3.98** | 3.72 | −2.02 |
|  | *qPh-1D* | E4 | 109.60 | *AX-109864377−AX-111484181* | **4.60** | 3.24 | −1.40 |
|  | *qPh-2B.1* | E1 | 62.60 | *AX-108751070−AX-108803655* | **8.57** | 5.10 | 2.55 |
|  |  | E2 | 68.80 | *AX-110102990−AX-109917562* | **8.38** | 5.13 | 2.60 |
|  |  | E3 | 48.40 | *AX-94904074−wPt-6932* | **3.12** | 4.23 | 1.63 |
|  |  | E4 | 46.80 | *AX-94904074−wPt-6932* | **6.67** | 5.44 | 1.81 |
|  |  | E5 | 44.20 | *Xwmc154−AX-109582821* | **4.53** | 4.12 | 2.16 |
|  |  | E6 | 47.60 | *AX-94904074−wPt-6932* | **3.33** | 3.34 | 1.91 |
|  |  | P | 49.20 | *AX-94904074−wPt-6932* | **5.95** | 4.78 | 2.00 |
|  | *qPh-2B.2* | E1 | 121.60 | *AX-110103130−AX-109464078* | 2.23 | 1.47 | 1.54 |
|  |  | E7 | 121.80 | *AX-110103130−AX-109464078* | **4.17** | 3.39 | 1.96 |
|  | *qPh-2D* | E1 | 123.20 | *Xme5em22−AX-111212405* | **3.51** | 2.03 | 1.61 |
|  |  | E7 | 120.00 | *AX-89716596−AX-110961575* | **8.87** | 6.25 | 2.26 |
|  |  | P | 119.60 | *AX-89716596−AX-110961575* | 2.70 | 1.76 | 1.21 |
|  | *qPh-3A.1* | E1 | 118.00 | *AX-94476859−AX-110453172* | **8.63** | 5.06 | 2.56 |
|  |  | E4 | 118.00 | *AX-94476859−AX-110453172* | **6.32** | 4.52 | 1.67 |
|  |  | E5 | 118.00 | *AX-94476859−AX-110453172* | 2.39 | 1.85 | 1.46 |
|  |  | E6 | 121.60 | *AX-109352439−Xwmc664* | 2.21 | 2.31 | 1.60 |
|  |  | E7 | 121.20 | *AX-109352439−Xwmc664* | **3.96** | 3.05 | 1.59 |
|  |  | P | 121.60 | *AX-109352439−Xwmc664* | **4.05** | 3.17 | 1.63 |
|  | *qPh-3A.2* | E2 | 140.60 | *AX-111127766−AX-111634399* | **6.03** | 3.45 | 2.14 |
|  | *qPh-3A.3* | E8 | 169.00 | *AX-94479536−AX-111167455* | **3.75** | 2.64 | 1.43 |
|  | *qPh-3D.1* | E1 | 92.40 | *AX-95248280−AX-110514763* | **9.60** | 5.69 | 2.68 |
|  |  | E2 | 94.20 | *AX-111163387−AX-108939123* | **4.87** | 2.74 | 1.89 |
|  |  | E3 | 94.20 | *AX-111163387−AX-108939123* | **10.39** | **13.07** | 2.86 |
|  |  | E4 | 94.40 | *AX-111163387−AX-108939123* | **11.16** | 8.56 | 2.27 |
|  |  | E5 | 92.20 | *AX-108889732−AX-95248280* | **7.63** | 6.77 | 2.77 |
|  |  | E6 | 94.00 | *AX-94785859−AX-111163387* | **9.33** | 8.82 | 3.11 |
|  |  | E7 | 94.60 | *AX-111163387−AX-108939123* | **11.43** | 8.29 | 2.60 |
|  |  | E8 | 94.80 | *AX-108939123−AX-109868592* | **15.85** | **12.64** | 3.13 |
|  |  | P | 92.40 | *AX-95248280−AX-110514763* | **13.79** | **10.20** | 2.92 |
|  | *qPh-3D.2* | E2 | 195.40 | *AX-109902667−AX-89343210* | **3.81** | 2.11 | −1.66 |
|  |  | E5 | 195.40 | *AX-109902667−AX-89343210* | 2.84 | 2.34 | −1.63 |
|  | *qPh-4A.1* | E4 | 141.80 | *Xgpw2331−AX-109313341* | **5.09** | 3.61 | −1.48 |
|  | *qPh-4A.2* | E2 | 205.60 | *Xbarc343−AX-110478858* | 2.03 | 1.05 | 1.26 |
|  |  | E8 | 205.60 | *Xbarc343−AX-110478858* | **5.80** | 4.12 | 1.92 |
|  | *qPh-4B.1* | E5 | 19.40 | *AX-111476002−Xmag4087* | **3.90** | 3.64 | −2.03 |
|  | *qPh-4B.2* | E1 | 39.40 | *Rht-B1−AX-108865778* | **18.76** | **13.18** | −4.10 |
|  |  | E2 | 39.20 | *Rht-B1−AX-108865778* | **24.65** | **18.00** | −4.86 |
|  |  | E3 | 40.80 | *AX-109390380−AX-109284839* | **13.98** | **18.50** | −3.43 |
|  |  | E4 | 39.40 | *Rht-B1−AX-108865778* | **20.46** | **17.66** | −3.27 |
|  |  | E5 | 41.00 | *AX-109390380−AX-109284839* | **16.30** | **16.01** | −4.28 |
|  |  | E6 | 41.00 | *AX-109390380−AX-109284839* | **28.03** | **33.87** | −6.12 |
|  |  | E7 | 41.00 | *AX-109390380−AX-109284839* | **27.37** | **24.49** | −4.50 |
|  |  | E8 | 40.20 | *Rht-B1−AX-108865778* | **28.52** | **27.62** | −4.66 |
|  |  | P | 41.00 | *AX-109390380−AX-109284839* | **34.01** | **33.50** | −5.32 |
|  | *qPh-5A* | E1 | 64.40 | *AX-109067598−AX-111015088* | **6.92** | 4.01 | −2.25 |
|  |  | E4 | 64.40 | *AX-109067598−AX-111015088* | **3.11** | 2.18 | −1.15 |
|  |  | E5 | 64.20 | *AX-109067598−AX-111015088* | **5.04** | 4.32 | −2.21 |
|  |  | E6 | 65.40 | *AX-108738850−wPt-3563* | **7.20** | 7.01 | −2.77 |
|  |  | E7 | 65.20 | *AX-108738850−wPt-3563* | **11.61** | 8.93 | −2.70 |
|  |  | E8 | 65.60 | *AX-108738850−wPt-3563* | **8.25** | 6.03 | −2.16 |
|  |  | P | 65.20 | *AX-108738850−wPt-3563* | **8.34** | 6.09 | −2.25 |
|  | *qPh-6B.1* | E1 | 109.00 | *Xsjz103−Xcnl113* | **14.67** | **9.46** | −3.46 |
|  |  | E2 | 108.80 | *AX-111619181−Xsjz91* | **12.90** | **8.47** | −3.33 |
|  |  | E3 | 116.60 | *AX-110400335−AX-109828920* | **11.46** | **14.72** | −3.04 |
|  |  | E4 | 109.00 | *Xsjz103−Xcnl113* | **19.22** | **16.74** | −3.18 |
|  |  | E5 | 116.80 | *AX-109828920−M218082-9* | **10.60** | **10.92** | −3.51 |
|  |  | E6 | 116.60 | *AX-110400335−AX-109828920* | **12.09** | **11.86** | −3.60 |
|  |  | E7 | 106.00 | *AX-111603010−AX-108960991* | **12.76** | **9.78** | −2.82 |
|  |  | E8 | 116.60 | *AX-110400335−AX-109828920* | **9.99** | **7.52** | −2.42 |
|  |  | P | 116.60 | *AX-110400335−AX-109828920* | **11.43** | **8.42** | −2.65 |
|  | *qPh-6B.2* | E1 | 143.80 | *AX-95659492−AX-110143566* | **3.52** | 1.94 | −1.57 |
|  |  | E4 | 144.20 | *AX-95659492−AX-110143566* | **6.19** | 4.44 | −1.64 |
|  | *qPh-6B.3* | E2 | 160.20 | *AX-111702680−Xcfe125* | **5.64** | 3.22 | −2.05 |
|  |  | E3 | 158.60 | *AX-111702680−Xcfe125* | **4.19** | 5.89 | −1.92 |
|  | *qPh-7D* | E5 | 153.40 | *AX-111529448−AX-110916001* | **3.76** | 3.14 | −1.89 |
|  |  | E8 | 148.20 | *AX-109873875−AX-89664808* | 2.44 | 2.43 | −1.37 |
| LTH | *qLth-1BL* | E3 | 2.60 | *Xwmc402.2−Xbarc187* | 2.60 | 3.45 | −1.59 |
|  | *qLth-2B.1* | E2 | 43.80 | *Xwmc154−AX-109582821* | 2.36 | 1.95 | 1.47 |
|  |  | P | 43.00 | *Xwmc154−AX-109582821* | 2.17 | 1.88 | 1.05 |
|  | *qLth-2B.2* | E1 | 90.00 | *AX-111021028−AX-109897993* | **3.34** | 2.59 | 1.77 |
|  |  | E2 | 95.00 | *AX-109338617−AX-109930013* | **4.16** | 3.37 | 1.96 |
|  |  | E3 | 90.40 | *AX-111037038−AX-110070301* | **3.47** | 4.53 | 1.75 |
|  |  | E4 | 94.00 | *AX-109284383−AX-110439888* | **5.39** | 7.27 | 2.37 |
|  |  | P | 94.00 | *AX-109284383−AX-110439888* | **4.59** | 3.89 | 1.55 |
|  | *qLth-2D.1* | E2 | 127.80 | *IN10−Xbarc228* | **3.43** | 2.76 | 1.74 |
|  |  | E7 | 123.20 | *Xme5em22−AX-111212405* | 2.00 | 2.94 | 1.33 |
|  | *qLth-2D.2* | E4 | 145.80 | *AX-94774356−AX-108762275* | **3.87** | 5.74 | 2.21 |
|  |  | E7 | 153.00 | *AX-94608041−AX-110411840* | 2.18 | 4.45 | 1.66 |
|  |  | P | 145.40 | *Xksum174.1−AX-94774356* | 2.34 | 1.89 | 1.13 |
|  | *qLth-3D.1* | E1 | 92.40 | *AX-95248280−AX-110514763* | **6.07** | 4.84 | 2.38 |
|  |  | E3 | 95.00 | *AX-109868592−AX-108842519* | **3.46** | 4.54 | 1.72 |
|  |  | E5 | 92.20 | *AX-108889732−AX-95248280* | 2.61 | 2.74 | 1.58 |
|  |  | E6 | 92.40 | *AX-95248280−AX-110514763* | **3.73** | 5.34 | 2.17 |
|  |  | E7 | 92.60 | *AX-110514763−AX-109165580* | **5.96** | 9.40 | 2.37 |
|  |  | E8 | 94.60 | *AX-111163387−AX-108939123* | **4.88** | 7.08 | 2.22 |
|  |  | P | 92.40 | *AX-95248280−AX-110514763* | **8.13** | 7.12 | 2.05 |
|  | *qLth-3D.2* | E1 | 199.80 | *AX-89662133−wPt-665570* | **3.43** | 2.95 | −1.86 |
|  |  | E6 | 195.40 | *AX-109902667−AX-89343210* | 2.02 | 2.70 | −1.55 |
|  |  | P | 197.60 | *AX-109366390−AX-111068370* | **3.83** | 3.21 | −1.38 |
|  | *qLth-3D.3* | E5 | 205.00 | *AX-111503420−AX-108920257* | **4.22** | 4.52 | −2.06 |
|  | *qLth-4A* | E5 | 196.00 | *AX-111284640−AX-95634684* | **3.56** | 3.77 | −1.85 |
|  |  | P | 195.80 | *AX-111123596−AX-111284640* | 2.22 | 1.74 | −1.01 |
|  | *qLth-4B* | E1 | 42.20 | *AX-110376140−AX-110455326* | **32.52** | **36.94** | −6.59 |
|  |  | E2 | 39.60 | *Rht-B1−AX-108865778* | **9.35** | 8.56 | −3.08 |
|  |  | E3 | 40.20 | *Rht-B1−AX-108865778* | **11.36** | **15.85** | −3.24 |
|  |  | E4 | 38.60 | *Rht-B1−Rht-B1* | **7.80** | **11.26** | −2.88 |
|  |  | E5 | 41.60 | *AX-109284839−AX-95145067* | **23.94** | **33.07** | −5.51 |
|  |  | E6 | 39.40 | *Rht-B1−AX-108865778* | **20.27** | **36.67** | −5.70 |
|  |  | E7 | 39.60 | *Rht-B1−AX-108865778* | **14.00** | **24.59** | −3.85 |
|  |  | E8 | 40.00 | *Rht-B1−AX-108865778* | **11.99** | **18.80** | −3.63 |
|  |  | P | 41.00 | *AX-109390380−AX-109284839* | **32.49** | **39.62** | −4.86 |
|  | *qLth-5A* | E1 | 59.20 | *AX-108793562−AX-94828773* | **3.31** | 2.59 | −1.74 |
|  |  | E5 | 66.00 | *wPt-3563−AX-111139819* | **4.28** | 4.88 | −2.11 |
|  |  | E7 | 64.20 | *AX-109067598−AX-111015088* | 2.08 | 2.96 | −1.33 |
|  | *qLth-5D.1* | E8 | 131.80 | *AX-89331886−Xgwm174* | **3.41** | 5.48 | −1.95 |
|  | *qLth-5D.2* | E1 | 251.40 | *AX-110587283−AX-110418893* | 2.09 | 1.73 | −1.42 |
|  |  | E5 | 254.60 | *AX-110418893−AX-110824131* | 2.78 | 2.92 | −1.63 |
|  | *qLth-6B.1* | E1 | 108.80 | *AX-111619181−Xsjz91* | **11.57** | **10.82** | −3.56 |
|  |  | E2 | 108.80 | *AX-111619181−Xsjz91* | **10.34** | **9.43** | −3.22 |
|  |  | E3 | 116.60 | *AX-110400335−AX-109828920* | **12.33** | **18.23** | −3.45 |
|  |  | E4 | 108.80 | *AX-111619181−Xsjz91* | **14.28** | **22.43** | −4.05 |
|  |  | E5 | 116.80 | *AX-109828920−M218082-9* | **4.72** | **6.15** | −2.36 |
|  |  | E6 | 110.20 | *Xwmc756−AX-110368657* | **6.55** | **9.78** | −2.95 |
|  |  | E7 | 116.80 | *AX-109828920−M218082-9* | **5.57** | **9.99** | −2.44 |
|  |  | E8 | 116.80 | *AX-109828920−M218082-9* | **5.98** | **10.35** | −2.68 |
|  |  | P | 116.80 | *AX-109828920−M218082-9* | **15.38** | **16.91** | −3.15 |
|  | *qLth-6B.2* | E1 | 132.40 | *wPt-1325−AX-110570347* | **5.98** | 4.90 | −2.45 |
|  |  | E2 | 132.40 | *wPt-1325−AX-110570347* | **4.50** | 3.70 | −2.07 |
|  |  | E3 | 132.40 | *wPt-1325−AX-110570347* | **3.08** | 4.07 | −1.67 |
|  |  | P | 132.40 | *wPt-1325−AX-110570347* | **4.45** | 3.84 | −1.54 |
|  | *qLth-6B.3* | E8 | 160.20 | *AX-111702680−Xcfe125* | **4.01** | 5.81 | −2.01 |
|  | *qLth-7A.1* | E1 | 84.20 | *AX-110737701−AX-89653008* | **3.42** | 2.64 | 1.76 |
|  | *qLth-7A.2* | E5 | 114.80 | *Xcfe260−AX-111052878* | **3.32** | 3.59 | 1.83 |
|  | *qLth-7A.3* | E1 | 240.00 | *AX-109898365−Xwmc809* | **4.08** | 3.18 | −1.95 |
|  | *qLth-7D* | E7 | 147.40 | *Xwmc264−AX-111477010* | **3.14** | 5.29 | −1.78 |
| SLT | *qSlt-1BL* | E3 | 6.60 | *Xme7em10.2−Xme7em19.1* | **3.84** | 9.44 | 1.71 |
|  | *qSlt-1D.1* | E7 | 49.60 | *AX-111540850−AX-109484943* | 2.25 | 4.22 | −1.19 |
|  |  | E8 | 45.00 | *AX-109278352−AX-110100033* | 2.25 | 5.03 | −1.43 |
|  | *qSlt-1D.2* | E7 | 55.40 | *AX-109906097−AX-110620313* | 2.11 | 6.30 | −1.49 |
|  |  | E8 | 55.80 | *AX-109906097−AX-110620313* | 2.34 | 5.46 | −1.49 |
|  | *qSlt-2A* | E3 | 25.80 | *AX-110601484−AX-111567412* | 2.45 | 5.59 | −1.28 |
|  |  | E4 | 27.40 | *AX-111567412−AX-110495160* | 2.30 | 4.65 | −1.25 |
|  | *qSlt-2B* | E5 | 120.00 | *AX-94898909−AX-111019647* | 2.10 | 4.00 | 1.42 |
|  | *qSlt-3A* | E2 | 168.00 | *AX-94479536−AX-111167455* | 2.31 | 4.59 | 0.94 |
|  |  | P | 167.60 | *AX-108768410−AX-94479536* | 2.11 | 3.84 | 0.66 |
|  | *qSlt-4A* | E2 | 152.00 | *AX-110037196−AX-110959122* | 2.15 | 4.17 | −0.90 |
|  |  | P | 150.80 | *AX-108966946−AX-110662742* | 2.26 | 4.15 | −0.68 |
|  | *qSlt-4B* | E8 | 72.00 | *Leaftype−AX-95659122* | **3.47** | 9.82 | −2.01 |
|  |  | P | 77.80 | *AX-109012953−AX-109408792* | 2.31 | 4.22 | −0.69 |
|  | *qSlt-4D.1* | E5 | 87.00 | *AX-95108577−AX-110545871* | **3.12** | 6.43 | −1.80 |
|  |  | E7 | 76.60 | *AX-109494858−AX-109913377* | 2.89 | 5.84 | −1.40 |
|  | *qSlt-4D.2* | E5 | 87.00 | *AX-95108577−AX-110545871* | **3.12** | 6.43 | −1.80 |
|  | *qSlt-5A* | E2 | 65.40 | *AX-108738850−wPt-3563* | **3.85** | 8.38 | −1.27 |
|  |  | E4 | 65.40 | *AX-108738850−wPt-3563* | 2.28 | 4.95 | −1.29 |
|  |  | E5 | 68.40 | *AX-109538871−AX-110612978* | 2.08 | 3.95 | −1.41 |
|  |  | E7 | 61.80 | *wPt-8226−Xwmc475* | 2.82 | 5.69 | −1.38 |
|  |  | P | 66.00 | *wPt-3563−AX-111139819* | **7.19** | **15.48** | −1.32 |
|  | *qSlt-5D* | E4 | 70.00 | *AX-109037061−AX-94687706* | **3.56** | 7.76 | 1.62 |
|  | *qSlt-6B.1* | E2 | 119.40 | *wPt-9952−AX-108988982* | **5.45** | **11.77** | −1.51 |
|  |  | E5 | 99.80 | *Xwmc737−Xswes199* | **3.62** | **7.46** | −1.94 |
|  |  | E7 | 106.00 | *AX-111603010−AX-108960991* | **11.01** | **25.92** | −2.95 |
|  |  | P | 106.00 | *AX-111603010−AX-108960991* | **4.76** | **9.69** | −1.04 |
|  | *qSlt-6B.2* | E4 | 141.40 | *AX-111466720−AX-109503661* | 2.84 | 7.21 | −1.56 |
|  | *qSlt-7A.1* | E5 | 112.60 | *AX-110911517−AX-110712776* | **3.84** | 7.79 | −1.98 |
|  | *qSlt-7A.2* | E2 | 174.20 | *AX-109914352−AX-109953544* | 2.15 | 5.74 | −1.06 |
|  |  | E7 | 179.20 | *AX-110934888−AX-94411296* | 2.21 | 5.88 | −1.40 |
|  | *qSlt-7A.3* | E1 | 239.00 | *AX-109311939−AX-109849040* | **3.52** | 8.41 | 1.49 |
| SLN | *qSln-1BL* | P | 31.20 | *wPt-2315−Ax-108850061* | **5.83** | 8.30 | −0.10 |
|  | *qSln-1D.1* | E2 | 45.20 | *AX-111540850−AX-109484943* | **3.29** | 4.92 | 0.12 |
|  | *qSln-1D.2* | E2 | 114.20 | *AX-108761985−AX-110335177* | **3.74** | 5.63 | −0.12 |
|  | *qSln-2A* | E5 | 56.40 | *AX-111079268−Xgwm636* | **4.14** | 9.48 | 0.21 |
|  | *qSln-2B.1* | E1 | 126.40 | *AX-111041164−AX-94403958* | **3.50** | 7.86 | 0.20 |
|  | *qSln-2B.2* | E5 | 152.80 | *AX-94450922−AX-109430774* | **4.39** | 6.52 | 0.17 |
|  | *qSln-2D* | E5 | 131.40 | *Xmag4089−Xmag4059* | **4.07** | 6.21 | 0.17 |
|  | *qSln-3D.1* | P | 98.40 | *AX-111109273−AX-111705267* | 2.93 | 4.03 | 0.07 |
|  | *qSln-3D.2* | E5 | 127.60 | *AX-110566282−AX-109340084* | **4.75** | 7.17 | −0.18 |
|  | *qSln-4A* | E3 | 196.80 | *AX-111284640−AX-95634684* | **3.40** | 7.95 | 0.18 |
|  |  | E5 | 196.20 | *AX-111284640−AX-95634684* | **3.99** | 5.94 | 0.17 |
|  |  | P | 191.60 | *AX-108872767−AX-110023758* | **3.31** | 4.88 | 0.08 |
|  | *qSln-4B.1* | E7 | 19.20 | *AX-111476002−Xmag4087* | 2.93 | 6.26 | −0.15 |
|  | *qSln-4B.2* | E1 | 38.20 | *Rht-B1−Rht-B1* | 2.47 | 4.81 | −0.13 |
|  | *qSln-4B.3* | E2 | 69.20 | *AX-111578547−AX-110596054* | **5.52** | 8.50 | −0.15 |
|  |  | E3 | 56.40 | *Xme16em26−AX-94855046* | 2.75 | 5.97 | −0.16 |
|  |  | E8 | 59.60 | *AX-108731280−AX-111609222* | 2.86 | 6.95 | −0.17 |
|  |  | P | 62.20 | *AX-110400686−AX-109879515* | **6.60** | 9.85 | −0.11 |
|  | *qSln-4D* | E4 | 85.20 | *AX-109376608−AX-110427760* | 2.65 | 5.95 | 0.17 |
|  | *qSln-5A* | E6 | 116.20 | *AX-111799065−AX-109903251* | 2.01 | 4.53 | −0.14 |
|  |  | E8 | 128.00 | *AX-110109453−AX-108799536* | 2.94 | 7.25 | −0.17 |
|  |  | P | 117.20 | *AX-110442597−AX-110456567* | **3.51** | 4.89 | −0.08 |
|  | *qSln-5B.1* | E5 | 116.00 | *AX-109019387−AX-94927526* | **7.38** | **11.29** | 0.23 |
|  | *qSln-5B.2* | E2 | 131.80 | *Xcfd156−AX-110622755* | **4.26** | 7.03 | 0.14 |
|  | *qSln-6B* | E2 | 106.00 | *AX-111603010−AX-108960991* | **19.24** | **36.52** | −0.32 |
|  |  | E7 | 106.00 | *AX-111603010−AX-108960991* | **6.13** | **14.04** | −0.23 |
|  |  | P | 106.00 | *AX-111603010−AX-108960991* | **3.86** | 5.45 | −0.08 |
|  | *qSln-7A.1* | E1 | 84.60 | *AX-110737701−AX-89653008* | **3.15** | 6.19 | −0.15 |
|  | *qSln-7A.2* | E1 | 239.00 | *AX-109311939−AX-109849040* | **3.12** | 6.15 | 0.15 |
|  | *qSln-7B* | E5 | 200.20 | *AX-110460106−AX-110935615* | **5.43** | 8.13 | 0.19 |
|  | *qSln-7D.1* | E6 | 147.60 | *AX-111477010−AX-108912914* | **3.19** | 7.66 | −0.18 |
|  | *qSln-7D.2* | E2 | 171.80 | *AX-95019577−AX-111989560* | **5.86** | 9.13 | −0.16 |
|  |  | P | 171.80 | *AX-95019577−AX-111989560* | **6.81** | 9.82 | −0.11 |
| SLU  16 | *qSlu-1BL* | P | 31.20 | *wPt-2315−Ax-108850061* | **4.38** | 6.94 | 0.01 |
|  | *qSlu-1D* | E2 | 114.20 | *AX-108761985−AX-110335177* | **3.53** | 5.71 | 0.01 |
|  | *qSlu-2A.1* | E4 | 45.20 | *AX-108750757−AX-108732889* | 2.42 | 6.00 | 0.02 |
|  | *qSlu-2A.2* | E5 | 56.40 | *AX-111079268−Xgwm636* | **3.18** | **11.38** | −0.02 |
|  | *qSlu-2B.1* | E2 | 105.00 | *AX-109923809−AX-111568967* | **3.18** | 5.13 | −0.01 |
|  | *qSlu-2B.2* | E1 | 126.40 | *AX-111041164−AX-94403958* | **3.73** | 8.00 | −0.02 |
|  | *qSlu-4A* | E3 | 197.20 | *AX-111284640−AX-95634684* | **3.13** | 8.51 | −0.01 |
|  |  | E5 | 196.20 | *AX-111284640−AX-95634684* | 2.85 | 6.42 | −0.02 |
|  |  | E8 | 190.40 | *AX-108872767−AX-110023758* | 2.31 | 5.66 | −0.02 |
|  |  | P | 191.80 | *AX-108872767−AX-110023758* | **3.05** | 5.04 | −0.01 |
|  | *qSlu-4B* | E2 | 69.20 | *AX-111578547−AX-110596054* | **5.67** | 9.42 | 0.01 |
|  |  | E8 | 59.60 | *AX-108731280−AX-111609222* | 2.38 | 5.85 | 0.02 |
|  |  | P | 62.60 | *AX-110400686−AX-109879515* | **5.34** | 8.72 | 0.01 |
|  | *qSlu-5A* | E8 | 128.00 | *AX-110109453−AX-108799536* | 2.63 | 7.04 | 0.02 |
|  |  | P | 117.20 | *AX-110442597−AX-110456567* | **3.45** | 5.46 | 0.01 |
|  | *qSlu-5B* | E2 | 131.80 | *Xcfd156−AX-110622755* | **3.01** | 5.21 | −0.01 |
|  | *qSlu-6B.1* | E2 | 106.00 | *AX-111603010−AX-108960991* | **5.65** | 9.54 | 0.01 |
|  |  | E7 | 106.00 | *AX-111603010−AX-108960991* | **6.70** | **18.43** | 0.04 |
|  |  | P | 106.00 | *AX-111603010−AX-108960991* | **4.99** | 8.14 | 0.01 |
|  | *qSlu-6B.2* | E1 | 131.00 | *AX-110494741−AX-108800056* | 2.70 | 7.14 | −0.02 |
|  | *qSlu-7A.1* | E1 | 84.60 | *AX-110737701−AX-89653008* | **3.39** | 6.50 | 0.01 |
|  | *qSlu-7A.2* | E1 | 239.00 | *AX-109311939−AX-109849040* | **3.36** | 6.44 | −0.01 |
|  | *qSlu-7D.1* | E6 | 147.60 | *AX-111477010−AX-108912914* | 2.91 | 7.02 | 0.02 |
|  | *qSlu-7D.2* | E2 | 171.80 | *AX-95019577−AX-111989560* | **4.69** | 7.76 | 0.01 |
|  |  | P | 171.80 | *AX-95019577−AX-111989560* | **5.22** | 8.38 | 0.01 |

LOD, Logarithm of odds; PVE, Phenotypic variance explained by the corresponding putative additive QTL; Add, additive effects of the corresponding putative additive QTL; positive values indicate Kenong 9204 alleles that increase the value of the corresponding trait, and, conversely, negative values indicate Kenong 9204 alleles that decrease it.

**Supplementary Table S11 Combined QTL analysis across environments for spike layer uniformity related traits detected in the KJ-RIL population**

| **QTL** | **LOD (Add)** | **LOD (A by E)** | Add (E1) | Add (E2) | Add (E3) | Add (E4) | Add (E5) | Add (E6) | Add (E7) | Add (E8) |
| --- | --- | --- | --- | --- | --- | --- | --- | --- | --- | --- |
| *qPh-1BL.1* | 23.35 | **6.22** | 0.43 | −1.73 | −0.25 | 0.83 | 0.22 | 0.74 | −0.40 | 0.16 |
| *qPh-1BL.2* | 0.97 | **5.74** | −0.75 | 1.81 | 0.07 | −0.35 | −0.39 | −0.38 | −0.02 | 0.02 |
| *qPh-1BL.3* | 4.85 | 0.30 | −0.17 | −0.03 | 0.40 | 0.20 | −0.52 | 0.07 | 0.06 | −0.02 |
| *qPh-1BL.4* | 6.68 | 0.90 | 0.14 | −0.16 | 0.20 | 0.23 | −0.58 | 0.42 | 0.01 | −0.26 |
| *qPh-1BL.5* | 11.42 | 1.54 | 0.14 | −0.01 | 0.53 | 0.19 | −0.10 | −1.12 | 0.23 | 0.16 |
| *qPh-1D* | 5.77 | **4.30** | 0.01 | −0.01 | −0.11 | −0.91 | 0.63 | −0.03 | 0.27 | 0.15 |
| *qPh-2B.1* | 19.45 | **3.80** | −0.67 | −0.60 | 0.19 | 0.35 | 0.68 | 0.67 | −0.33 | −0.29 |
| *qPh-2B.2* | 5.97 | 1.72 | 0.00 | −0.05 | −0.60 | −0.16 | 0.29 | −0.15 | 0.84 | −0.18 |
| *qPh-2D* | 5.40 | **6.02** | −0.69 | 0.06 | −0.18 | −0.21 | −0.13 | −0.10 | 1.59 | −0.34 |
| *qPh-3A.1* | 11.25 | **6.50** | 1.42 | −0.64 | −0.51 | 0.68 | 0.61 | −0.54 | −0.55 | −0.47 |
| *qPh-3A.2* | 3.71 | **3.79** | −0.25 | −0.55 | 0.93 | 0.09 | 0.81 | −0.28 | −0.50 | −0.26 |
| *qPh-3A.3* | 2.33 | **3.84** | −0.16 | −0.71 | −0.18 | 0.27 | 0.31 | −0.54 | 0.06 | 0.95 |
| *qPh-3D.1* | 12.47 | **16.93** | −0.93 | −0.78 | 1.53 | 1.17 | 1.92 | −0.97 | −0.95 | −0.98 |
| *qPh-3D.2* | 9.08 | 1.57 | −0.09 | −0.87 | 0.56 | 0.59 | −0.89 | 0.36 | 0.22 | 0.11 |
| *qPh-4A.1* | 6.36 | **3.93** | 0.32 | 0.09 | 0.10 | −0.98 | 0.25 | 0.04 | 0.14 | 0.03 |
| *qPh-4A.2* | 10.05 | 1.35 | −0.29 | 0.03 | −0.25 | −0.22 | 0.16 | 0.09 | −0.12 | 0.61 |
| *qPh-4B.1* | 3.48 | 0.62 | 0.39 | −0.06 | 0.18 | 0.26 | −0.81 | −0.18 | −0.08 | 0.31 |
| *qPh-4B.2* | 86.40 | **50.57** | −3.65 | −5.26 | 2.80 | −0.47 | 2.83 | 2.75 | 2.71 | −1.70 |
| *qPh-5A.1* | 24.21 | **10.32** | 1.34 | 0.50 | 1.45 | 0.65 | −0.97 | −1.53 | −1.00 | −0.43 |
| *qPh-6B.1* | 21.69 | **22.15** | −2.02 | 0.98 | 0.82 | −1.81 | 0.38 | 1.40 | 0.98 | −0.73 |
| *qPh-6B.2* | 4.72 | **5.19** | 0.58 | 0.19 | −0.15 | −1.10 | 0.03 | 0.16 | −0.17 | 0.47 |
| *qPh-6B.3* | 4.85 | **3.62** | 0.17 | 0.08 | −1.02 | 0.37 | 0.24 | 0.08 | −0.12 | 0.21 |
| *qPh-7D* | 4.38 | 0.79 | 0.09 | 0.13 | 0.61 | 0.09 | −0.80 | −0.04 | −0.01 | −0.08 |
| *qLth-1BL* | 3.67 | 1.88 | 0.53 | −0.07 | −0.73 | 0.05 | 0.08 | 0.63 | −0.25 | −0.23 |
| *qLth-2B.1* | 6.48 | 1.16 | 0.05 | 0.51 | −0.27 | −0.34 | 0.33 | −0.43 | 0.12 | 0.04 |
| *qLth-2B.2* | 4.93 | **3.47** | −0.31 | −0.71 | −0.32 | 1.59 | −0.42 | −0.28 | 0.05 | 0.40 |
| *qLth-2D.1* | 3.93 | 1.31 | 0.37 | −0.66 | 0.14 | −0.35 | −0.16 | 0.17 | 0.42 | 0.06 |
| *qLth-2D.2* | 7.35 | **2.56** | 0.00 | −1.22 | −0.27 | 1.02 | 0.02 | 0.05 | 0.15 | 0.24 |
| *qLth-3D.1* | 9.99 | **7.37** | 1.26 | −0.31 | −1.16 | 0.11 | −1.14 | 0.97 | 1.18 | −0.90 |
| *qLth-3D.2* | 8.30 | 1.38 | 0.92 | −0.11 | 0.21 | 0.13 | −0.79 | −0.38 | −0.10 | 0.12 |
| *qLth-3D.3* | 7.04 | 1.61 | −0.79 | 0.01 | −0.07 | −0.06 | 0.61 | 0.24 | 0.02 | 0.06 |
| *qLth-4A* | 7.67 | 1.71 | 0.53 | 0.48 | −0.14 | 0.18 | −1.13 | 0.26 | 0.08 | −0.26 |
| *qLth-4B* | 58.40 | **13.58** | 2.37 | −0.18 | −0.50 | 0.03 | 2.51 | −2.49 | −0.92 | −0.81 |
| *qLth-5A* | 5.68 | 1.48 | 0.42 | 0.36 | 0.38 | 0.59 | −0.65 | −0.55 | −0.40 | −0.16 |
| *qLth-5D.1* | 3.69 | 2.03 | 0.27 | −0.05 | 0.05 | 0.31 | 0.05 | 0.52 | −0.07 | −1.09 |
| *qLth-5D.2* | 2.57 | **3.07** | −0.76 | −0.34 | 0.82 | 0.11 | −0.65 | −0.47 | 0.46 | 0.84 |
| *qLth-6B.1* | 17.10 | **8.36** | 0.99 | 1.14 | −1.55 | 0.80 | −0.54 | 0.71 | −0.70 | −0.85 |
| *qLth-6B.2* | 2.35 | 0.97 | 0.32 | 0.43 | 0.42 | −0.29 | 0.10 | −0.45 | −0.28 | −0.25 |
| *qLth-6B.3* | 6.73 | **2.53** | −0.10 | 0.47 | 0.10 | 0.45 | 0.81 | −0.39 | −0.28 | −1.06 |
| *qLth-7A.1* | 1.80 | 2.48 | 1.28 | −0.35 | −0.17 | −0.06 | 0.29 | −0.19 | −0.44 | −0.36 |
| *qLth-7A.3* | 1.84 | **3.60** | −1.48 | −0.31 | 0.21 | 0.61 | 0.71 | −0.03 | −0.01 | 0.30 |
| *qLth-7D* | 2.48 | 1.06 | 0.40 | 0.11 | −0.20 | −0.29 | −0.25 | 0.62 | 0.01 | −0.39 |
| *qSlt-1BL* | 0.31 | **3.95** | −0.43 | −0.23 | 1.28 | 0.26 | 0.19 | −0.17 | −0.35 | −0.54 |
| *qSlt-1D.2* | 2.66 | 1.73 | −0.37 | 0.29 | 0.28 | 0.08 | 0.14 | 0.34 | −0.18 | −0.57 |
| *qSlt-2A* | 2.31 | **5.57** | −0.31 | −0.10 | −0.72 | −0.76 | 1.11 | 0.78 | 0.43 | −0.43 |
| *qSlt-2B* | 1.96 | 1.30 | 0.10 | −0.22 | −0.15 | −0.70 | 0.90 | 0.35 | −0.18 | −0.10 |
| *qSlt-3A* | 4.46 | 1.99 | 0.19 | 0.25 | −0.05 | 0.04 | −0.54 | 0.20 | −0.41 | 0.32 |
| *qSlt-4A* | 4.00 | 1.33 | −0.14 | −0.17 | 0.36 | 0.21 | 0.39 | −0.90 | 0.30 | −0.05 |
| *qSlt-4B* | 4.28 | 2.33 | 0.00 | 0.76 | 0.60 | −0.10 | −0.04 | −0.46 | 0.26 | −1.03 |
| *qSlt-4D.1* | 0.83 | **3.24** | 0.18 | 0.10 | 0.30 | 0.53 | -0.03 | 0.25 | -0.94 | -0.39 |
| *qSlt-4D.2* | 1.76 | **3.58** | −0.19 | −0.02 | 0.31 | 0.85 | −1.37 | 0.46 | 0.36 | −0.41 |
| *qSlt-5A* | 10.21 | **2.65** | −0.08 | −0.25 | 0.18 | −0.18 | −0.04 | −0.64 | 1.00 | 0.00 |
| *qSlt-5D* | 0.52 | **3.63** | −0.60 | −0.31 | 0.13 | 1.38 | 0.00 | −0.22 | −0.31 | −0.08 |
| *qSlt-6B.1* | 5.74 | **8.40** | 0.22 | 0.40 | 0.13 | 0.71 | 0.43 | −0.16 | −1.67 | −0.06 |
| *qSlt-7A.1* | 3.53 | 1.87 | 0.48 | 0.20 | 0.11 | 0.25 | −1.31 | −0.55 | 0.54 | 0.29 |
| *qSlt-7A.3* | 4.35 | **2.54** | 0.83 | −0.04 | −0.11 | −0.72 | 0.27 | 0.20 | −0.41 | −0.02 |
| *qSln-1BL* | 4.20 | 1.04 | −0.03 | 0.01 | 0.00 | 0.03 | 0.01 | −0.05 | 0.00 | 0.04 |
| *qSln-1D.1* | 0.01 | **4.31** | −0.01 | 0.10 | 0.01 | −0.01 | 0.04 | −0.02 | −0.03 | −0.08 |
| *qSln-1D.2* | 0.19 | **3.66** | 0.01 | −0.10 | 0.02 | 0.00 | 0.05 | −0.04 | 0.03 | 0.03 |
| *qSln-2A* | 0.86 | **4.37** | −0.06 | 0.01 | 0.00 | −0.09 | 0.12 | 0.01 | 0.02 | −0.01 |
| *qSln-2B.1* | 1.04 | 2.17 | 0.07 | 0.02 | 0.03 | −0.05 | 0.00 | −0.02 | −0.02 | −0.03 |
| *qSln-2B.2* | 1.41 | **3.58** | −0.01 | −0.02 | 0.04 | 0.00 | 0.13 | −0.03 | −0.06 | −0.06 |
| *qSln-3D.1* | 3.41 | 0.61 | −0.01 | −0.02 | 0.04 | 0.03 | 0.02 | −0.01 | −0.02 | −0.04 |
| *qSln-3D.2* | 0.95 | **3.44** | 0.00 | 0.02 | 0.01 | 0.06 | −0.14 | −0.01 | 0.03 | 0.02 |
| *qSln-4A* | 3.54 | **6.10** | −0.02 | −0.07 | 0.11 | −0.09 | 0.11 | −0.07 | −0.02 | 0.04 |
| *qSln-4B.1* | 1.27 | **2.89** | 0.08 | 0.04 | 0.03 | −0.02 | 0.00 | −0.03 | −0.08 | −0.03 |
| *qSln-4B.2* | 3.08 | 1.97 | −0.06 | 0.03 | 0.08 | 0.02 | −0.02 | −0.04 | 0.02 | −0.01 |
| *qSln-4B.3* | 1.78 | **4.99** | 0.03 | −0.11 | 0.07 | −0.05 | −0.01 | 0.00 | 0.01 | 0.04 |
| *qSln-4D* | 1.12 | **4.09** | −0.03 | 0.01 | 0.03 | 0.13 | −0.10 | 0.05 | −0.05 | −0.05 |
| *qSln-5A* | 3.36 | 1.19 | 0.00 | 0.04 | 0.00 | 0.02 | 0.01 | 0.00 | 0.02 | −0.09 |
| *qSln-5B.1* | 2.61 | **5.56** | −0.03 | −0.04 | −0.02 | −0.08 | 0.17 | 0.01 | −0.04 | 0.03 |
| *qSln-5B.2* | 2.75 | **3.60** | 0.03 | 0.08 | −0.01 | −0.01 | −0.05 | −0.02 | −0.05 | 0.03 |
| *qSln-6B* | 11.29 | **17.59** | 0.06 | −0.21 | 0.06 | 0.13 | 0.01 | 0.00 | −0.09 | 0.04 |
| *qSln-7A.2* | 2.23 | **2.70** | 0.10 | 0.01 | 0.01 | −0.06 | −0.02 | 0.01 | −0.03 | −0.01 |
| *qSln-7D.2* | 7.44 | **3.21** | 0.00 | −0.07 | −0.01 | 0.03 | 0.02 | 0.01 | 0.02 | 0.00 |
| *qSlu-1BL* | 3.50 | 1.74 | 0.00 | 0.00 | 0.00 | 0.00 | 0.00 | 0.01 | 0.00 | 0.00 |
| *qSlu-1D* | 0.44 | **3.35** | 0.00 | 0.01 | 0.00 | 0.00 | 0.00 | 0.00 | 0.00 | 0.00 |
| *qSlu-2A.1* | 0.05 | **4.56** | 0.01 | 0.00 | 0.00 | 0.01 | 0.00 | 0.00 | −0.01 | 0.00 |
| *qSlu-2A.2* | 1.21 | **3.81** | 0.01 | 0.00 | 0.00 | 0.01 | −0.01 | 0.00 | −0.01 | 0.00 |
| *qSlu-2B.1* | 0.51 | **3.55** | 0.00 | −0.01 | 0.00 | 0.01 | 0.00 | 0.00 | 0.00 | 0.00 |
| *qSlu-2B.2* | 0.71 | **2.73** | −0.01 | 0.00 | 0.00 | 0.00 | 0.00 | 0.00 | 0.00 | 0.00 |
| *qSlu-4A* | 1.46 | **5.00** | 0.00 | 0.00 | −0.01 | 0.01 | −0.01 | 0.00 | 0.00 | 0.01 |
| *qSlu-4B* | 2.09 | **5.79** | 0.00 | 0.01 | 0.00 | 0.00 | 0.00 | 0.00 | 0.00 | −0.01 |
| *qSlu-5A* | 4.86 | 2.17 | 0.00 | −0.01 | 0.00 | −0.01 | 0.00 | 0.00 | 0.00 | 0.02 |
| *qSlu-5B* | 2.50 | **3.06** | 0.00 | 0.00 | 0.00 | 0.00 | 0.00 | 0.00 | 0.01 | −0.01 |
| *qSlu-6B.1* | 9.73 | **6.26** | 0.00 | 0.00 | −0.01 | −0.01 | 0.00 | 0.00 | 0.02 | 0.00 |
| *qSlu-6B.2* | 0.02 | **3.80** | −0.01 | 0.00 | 0.00 | 0.01 | 0.00 | 0.00 | 0.00 | 0.00 |
| *qSlu-7A.2* | 2.95 | **2.54** | −0.01 | 0.00 | 0.00 | 0.00 | 0.00 | 0.00 | 0.00 | 0.00 |
| *qSlu-7D.2* | 5.50 | **3.40** | 0.00 | 0.00 | 0.00 | −0.01 | 0.00 | 0.00 | 0.00 | 0.00 |

LOD (Add), Logarithm of odds for the QTL with additive effects; LOD (A by E), Logarithm of odds for the QTL with additive-by-environment interaction effects; Add (E), additive effects of the corresponding putative additive QTL in the corresponding environment; positive values indicate Kenong 9204 alleles that increase the value of the corresponding trait, and, conversely, negative values indicate Kenong 9204 alleles that decrease it. LOD (A by E) by *bold* typeface indicated that a QTL showed significant additive-by-environment interaction effects.
